# How are children's perceptions of the home environment associated with a general psychopathology factor across childhood?

**DOI:** 10.1111/jcpp.70046

**Published:** 2025-09-08

**Authors:** Jack K. Nejand, Margherita Malanchini, Ivan Voronin, Thalia C. Eley, Kaili Rimfeld

**Affiliations:** ^1^ Institute of Psychiatry, Psychology and Neuroscience King's College London London UK; ^2^ School of Biological and Behavioural Sciences Queen Mary, University of London London UK; ^3^ Social, Genetic and Developmental Psychiatry Centre Institute of Psychiatry, Psychology and Neuroscience, King's College London London UK; ^4^ École de Psychologie Université Laval Quebec City QC Canada; ^5^ Department of Psychology, Royal Holloway University of London Surrey UK

**Keywords:** General factor of psychopathology, p‐factor, childhood psychopathology, twin analysis, home environment, cross‐lag model

## Abstract

**Background:**

Comorbidity and heterogeneity in psychiatric disorders may stem from a general psychopathology (p) factor influenced by both genetic and environmental factors. Although the relative contributions of these influences on psychopathology are established, the longitudinal associations between the p‐factor and specific environmental exposures and the aetiology of these associations across development are not well understood. Here, we examine whether twin‐rated home environment contributes to changes in the p‐factor over time or, conversely, whether the p‐factor influences twin‐rated home environment, reflecting potential evocative gene–environment processes.

**Methods:**

Data were obtained from the Twins Early Development Study (TEDS). Cross‐lagged panel analyses were conducted separately to ascertain the direction of associations between parent‐rated p, twin‐rated p, and twin‐rated home environment (chaos at home and parental discipline) at ages 9, 12, and 16 (*N* = 6,213). Biometric autoregressive cross‐lagged twin models were used to assess the aetiology of these associations, and MZ differences analyses were used to control for familial effects.

**Results:**

Both parent‐rated and twin‐rated p‐factor and twin‐rated home environment were stable over time, although the twin‐rated p‐factor (*r* = .44 [0.42, 0.46]–.40 [0.37, 0.41]) was more variable than the parent‐rated p‐factor (*r* = .72 [0.71, 0.74]–.63 [0.61, 0.64]). Twin‐rated home environment was more variable than p‐factor in all cross‐lagged models (phenotypic and MZ differences). Small, significant bidirectional associations were found between the p‐factor and twin‐rated home environment, with stronger cross‐lagged paths from the p‐factor to the twin‐rated home environment than vice versa. These longitudinal associations persisted over time, though attenuated for parent‐rated p‐factor. Genetic analyses revealed that bidirectional cross‐lagged paths were largely explained by shared environmental factors, with a smaller proportion explained by genetic factors. This pattern of results was confirmed in MZ difference analyses.

**Conclusions:**

Our findings suggest a dynamic and bidirectional relationship between p‐factor and twin‐rated home environment across development, predominantly influenced by shared environmental factors. Changes in one can affect the other, highlighting the complexity of psychopathology's environmental influences. This underscores the need for further investigation into gene–environment interplay to inform prevention and intervention strategies for psychopathology.

## Introduction

The prevalence of psychopathology amongst young people continues to rise, with 1‐in‐6 UK‐based 6–19‐year‐olds experiencing identifiable mental health needs in 2021, up from 1‐in‐9 in 2017 (NHS Digital, 2021). Furthermore, a 2017 report identified 5% of British under‐20s met criteria for multiple mental health disorders (NHS Digital, 2017), with rates likely to be much higher today. The overlap between disorders in adults roughly conforms to a ‘rule of 50%’, meaning that half of those with one disorder are likely to meet diagnostic criteria for a second disorder, and half of this group will meet diagnostic criteria for a third and so on (Caspi et al., 2014).

Recent research suggests a single component may explain such high rates of comorbidity, labelled a general psychopathology factor or ‘p‐factor’ (Caspi et al., 2014; Caspi & Moffitt, 2018; Lahey et al., 2012). The first principal component of mental health symptoms, indicative of a p‐factor, accounts for a significant proportion of variance in psychiatric disorders– ranging from 40% to 50% across different data types (Allegrini et al., 2020). Higher p‐factor scores are associated with higher rates of psychiatric symptoms and thus associated with life impairment, such as suicide attempts, reliance on welfare, and violence convictions (Caspi et al., 2014). Higher p‐factor is also linked to lower cognitive functioning longitudinally (Von Stumm, Malanchini, & Fisher, 2024).

The consistent heritability of and high genetic correlations between all psychiatric disorders supports the notion that the heritability of psychiatric disorders is largely explained by a single shared genetic factor (Kendler et al., 2011; Smoller et al., 2013). Twin studies report that genetic factors explain 20%–80% of p‐factor longitudinally (Selzam, Coleman, Caspi, Moffitt, & Plomin, 2018), and genetic factors also account for the relative stability across development (Allegrini et al., 2020). Genomic studies show consistent evidence of heritability for p‐factor (Grotzinger et al., 2022; Keser et al., 2023; Selzam et al., 2018). However, these leave a moderate proportion of the individual differences in p‐factor attributable to environmental influences (e.g. 20%–52%; Allegrini et al., 2020).

The home environment provides a meaningful context whereby the environmental impact on development can be directly observed. ‘Household chaos’, defined by excessive noise, overcrowding, and an absence of routine or structure (Jaffee, Hanscombe, Haworth, Davis, & Plomin, 2012), has often been associated with psychopathological outcomes. Children growing up in chaotic households have been found more likely to exhibit less behavioural control (*r* ~ .38) (Vrijhof, van der Voort, van IJzendoorn, & Euser, 2018), more disruptive behaviour (*r* ~ .25) (Jaffee et al., 2012), higher rates of depression (*r* ~ .30) (Tucker, Sharp, Van Gundy, & Rebellon, 2018) and have poorer academic achievement (*r* ~ −.26) (Hanscombe, Haworth, Davis, Jaffee, & Plomin, 2011) and fewer social skills (*r* ~ −.07) (Bobbitt & Gershoff, 2016).

Less is known about the longitudinal associations between perceptions of the home environment, how this changes over time, and its impact on psychopathological outcomes. Evidence suggests that adolescents' perceptions of their home environment may have a stronger link to their mental health than parental reports (Human, Dirks, DeLongis, & Chen, 2016). However, the direction of these associations between mental health and twin‐rated home environment remains unclear. The perceptions of household chaos are often considered to be risk factors for children's poor developmental outcomes; it may also be a result of children's behaviour (Jaffee et al., 2012). Similarly, parenting behaviours, such as maternal negativity and harsh discipline, are associated with increased internalising and externalising problems in children (Chen, Deater‐Deckard, & Bell, 2014), but it is possible that these difficulties shape parental behaviour over time. This article has therefore included twin‐rated measures of both ‘household chaos’ and ‘parental discipline' to define ‘home environment.’ Despite increasing recognition of the bidirectional relationships between home environment and psychopathology, there is limited research examining these associations longitudinally, particularly using genetically sensitive designs. It is important to understand the bidirectional relationship between the home environment and psychopathology using genetically sensitive study designs; it allows us to study whether environmental factors reflect underlying genetic predispositions or actively shape developmental outcomes. Establishing the direction of these associations is crucial for determining whether interventions targeting the home environment can meaningfully alter psychopathological trajectories, or whether the influence primarily flows from psychopathology to the home environment, suggesting that p‐factor shapes environmental experiences rather than the reverse.

The concept of heritability in environmental measures, such as ‘household chaos,’ suggests that children's experiences of their environment are not independent of their genetic predispositions (Hanscombe et al., 2011). This introduces the concept of gene–environment correlation (rGE), where genetic factors co‐occur with certain environmental exposures, in turn affecting psychopathological development (Agnew‐Blais et al., 2022; Knafo & Jaffee, 2013; Knopik, Neiderhiser, DeFries, & Plomin, 2017). For example, household chaos may reflect a passive rGE, where parents with a genetic predisposition for mental health difficulties create more chaotic home environments, exposing children to both inherited risk and environmental instability. Evocative rGE suggests that children's genetically influenced traits, for example, hyperactivity, can elicit disorganised parenting and influence household chaos. Additionally, active rGE may play a role as children grow, as they may seek out or perpetuate environments that align with their genetic predispositions (Avinun & Knafo, 2014; Eley, Napolitano, Lau, & Gregory, 2010; Knopik et al., 2017). In line with this broad view, we use the term psychopathology here to include neurodevelopmental conditions such as autism alongside common emotional and behavioural disorders.

Our understanding of how genetic and environmental factors contribute to psychopathology is still developing. Here, we study the relationship between the twin‐rated home environment, measured as parental discipline and chaos in the household, and psychopathology, aiming to clarify how they may influence each other. We investigate these associations over time, offering new insights into the role of the home environment in shaping and being shaped by psychopathology. Although TEDS includes multiple home environment measures, we focus on chaos and parental discipline as they have consistently been measured across development, allowing for a robust longitudinal analysis. The moderate heritability of p‐factor and twin‐rated home environment highlights the importance of using genetically informative designs to understand their relationship better. Doing so longitudinally also allows us to study the extent and direction of associations between these constructs, overcoming the limitations of previous cross‐sectional studies. Genetic cross‐lagged models were also calculated to estimate the respective variance explained by additive genetic (A), shared environmental (C), and nonshared environmental (E) effects. Finally, an MZ differences design was used to control both A and C effects, allowing us to examine differences in twin‐rated home environment and psychopathology within MZ twin pairs. This provides further insight into how siblings raised in the same household may perceive and report their environment differently, clarifying the contribution of nonshared environmental influences. The present study was registered on the Open Science Framework (https://osf.io/2xgtd/) with the following aims:

1. To explore the association between twin‐rated home environment and p‐factor, longitudinally from age 9 to 16, and the aetiology of these associations.
2. To test the stability of p‐factor and twin‐rated home environment across childhood using both parent and child report.
3. To identify how these associations are affected when controlling for genetic and shared environmental factors using an autoregressive cross‐lagged twin model and MZ differences design.

## Methods

### Participants

The present study utilises data from the Twins Early Development Study (TEDS), a longitudinal twin study that recruited over 16,000 twin pairs born in England and Wales between 1994 and 1996. Despite some attrition, more than 10,000 twin pairs remain actively involved in the study. This cohort remains representative of the wider English and Welsh population in terms of ethnic and socioeconomic factors across waves for their birth cohort (Lockhart et al., 2023). Rich behavioural data have been collected from the twins, their parents, and teachers over three decades.

The present study used all available longitudinal data from 18,548 participants (9,274 twin pairs), with up to 6,212 pairs completing any one measure. The missing data were especially evident from age 16 data collection, as data were collected only from 2 out of 4 birth cohorts during this data collection wave. Full information maximum likelihood was used to account for missing data, assuming data were missing at random and including all cases with partial data. Using both members of the twin pair in any analysis artificially inflates the sample size, so a random member of each pair was selected for the phenotypic analyses. This sample was 50.9% female, and 34.4% were from monozygotic pairs. Analyses were conducted on measures collected at ages 9 (*M* = 9.02, *SD* = 0.29), 12 (*M* = 11.31, *SD* = 0.72), and 16 (*M* = 16.32, *SD* = 0.68) from twins themselves (p‐factor and home environment measures) and from their parents (p‐factor measures only). These ages were chosen to capture the transition from middle childhood into adolescence, and the same home environment measures were available at each, enabling longitudinal analyses.

### Measures

The present study utilises measures to reflect p‐factor described in a recent TEDS study (Allegrini et al., 2020). This approach is hypothesis‐free to capture as many relevant domains as possible. It includes measures not always used in the prior p‐factor literature, such as those for developmental disorders (e.g. autism traits). Table 1 outlines the measures used to capture p‐factor and home environment composites. More information about the measures used can be found in the TEDS Data Dictionary (https://www.teds.ac.uk/datadictionary/home.htm). All p‐factor measures are well‐validated and have previously demonstrated sound psychometric properties, with moderate to high levels of internal consistency (all Cronbach's alpha > .6). Different measures were used at different waves to capture parallel constructs at each wave whilst maintaining developmental appropriateness. The home environment measures (CHAOS and parental discipline, Table 1) were chosen as they have been consistently administered at each selected wave.

**Table 1:** Summary of measures included to calculate p‐factor and home environment composites

Wave age	p‐factor measures		Home environment measures (all twin‐rated)
	Parent‐rated	Twin‐rated	
9	SDQ Emotional Problems SDQ Prosocial SDQ Hyperactivity SDQ Conduct SDQ Peer Problems CAST Social CAST Non‐Social CAST Communication APSD Impulsivity APSD Narcissism RPAQ Reactive Aggression RPAQ Proactive Aggression	SDQ Emotional Problems SDQ Prosocial SDQ Hyperactivity SDQ Conduct SDQ Peer Problems CAST Social CAST Non‐Social CAST Communication	CHAOS Parental discipline
12	SDQ Emotional Problems SDQ Prosocial SDQ Hyperactivity SDQ Conduct SDQ Peer Problems MFQ CAST Social CAST Non‐Social CAST Communication APSD Impulsivity APSD Callous‐Unemotional APSD Narcissism CBRS Inattention CBRS Hyperactivity	SDQ Emotional Problems SDQ Prosocial SDQ Hyperactivity SDQ Conduct SDQ Peer Problems MFQ	CHAOS Parental discipline
16	SDQ Emotional Problems SDQ Prosocial SDQ Hyperactivity SDQ Conduct AQ Attention to Detail AQ Imagination AQ Attention Switching AQ Social MFQ CBRS Inattention CBRS Hyperactivity ICUT Uncaring ICUT Unemotional ICUT Callous	SDQ Emotional Problems SDQ Prosocial SDQ Hyperactivity SDQ Conduct SDQ Peer Problems MFQ AQ Attention to Detail AQ Social	CHAOS Parental discipline

### General factor of psychopathology

To capture the p‐factor, the following constructs were used: *ADHD* (Conners' Comprehensive Behavioural Rating Scale [CBRS]; Conners, 1970), *aggression* (Reactive‐Proactive Aggression Questionnaire [RPAQ]; Raine et al., 2006; Eley et al., 2003), *autism traits* (Childhood Autism Spectrum Test [CAST]; Scott, Baron‐Cohen, Bolton, & Brayne, 2002; Autism Quotient [AQ]; Baron‐Cohen, Wheelwright, Skinner, Martin, & Clubley, 2001), *callous‐unemotional traits* (Inventory for the Callous‐Unemotional Scale [ICUT]; Frick, 2004), *depression* (Moods and Feelings Questionnaire [MFQ]; Angold, Costello, Pickles, & Winder, 1987), *emotional problems* (Strengths and Difficulties Questionnaire [SDQ – Anxiety subscale]; Goodman, 1997), *hyperactivity* (SDQ – hyperactivity subscale), *peer problems* (SDQ – peer problems subscale), *prosocial behaviour* (SDQ – prosocial subscale, reversed) and *psychopathic tendencies* (Anti‐Social Process Screening Device [APSD]; Frick & Hare, 2001).

### Home environment

To capture twin‐rated home environment, a composite was calculated using the following constructs at all three waves: *household chaos* (Confusion, Hubbub, and Order Scale [CHAOS]; Matheny Jr, Wachs, Ludwig, & Phillips, 1995) and *parental discipline* (derived from Deater‐Deckard, Dodge, Bates, & Pettit, 1998). These measures were chosen as they have been consistently administered at each selected wave. A summary of the measures administered at each wave can be found in Table 1. Both household chaos and parental discipline were twin‐rated and captured their perceptions of the home environment. The two measures correlate at *r* ~ .3, indicating a moderate association. A composite score was used in the main analyses to provide a broader measure of environmental influences. Additionally, analyses were conducted separately for household chaos and parental discipline to examine their individual contributions (See Appendix S1).

Data for each wave in the present study were collected from twins and parents by TEDS via a test booklet, with an additional well‐being questionnaire administered digitally to twins at 16. The dataset was exported to an SPSS (IBM Statistical Product and Service Solutions 26) file. Measures were adjusted for age and sex, and descriptive statistics were generated. Correlations and cross‐lagged panel model analyses were conducted using R version 3.6.3 (R Core Team, 2020). The following packages were in the main analyses: lavaan (Rosseel, 2012), psych (v2.1.3; Revelle, 2020), ggplot2 (Wickham, 2016). The following packages were used for cross‐lagged analyses: devtools (v2.4.0; Wickham, Hester, & Chang, 2021), tidyverse (Wickham et al., 2019), OpenMX (v2.0; Neale et al., 2016; Pritikin, Hunter, & Boker, 2015; Hunter, 2018), openxlsx (v4.2.3; Schauberger & Walker, 2020), mlth.data.frame (v1.2; Voronin, 2020), and TwinAnalysis (v0.2.0; Voronin, 2021).

### Statistical analysis

#### Descriptive statistics and sex differences between measures

Means and standard deviations were calculated for each phenotypic variable (see Table S1). Sex differences between variables were also calculated using ANOVA.

#### Extracting p‐factor and home environment composites via factor analyses

To model p‐factor, we conducted confirmatory factor analyses (CFA) using the lavaan package with full information maximum likelihood to account for missing data. All mental health measures showed high loadings on the first factor, indicating a coherent structure for p‐factor, as previously demonstrated by Allegrini et al. (2020). The primary goal of using CFA was not to identify the best‐fitting model for developmental psychopathology but rather to use it as a data reduction technique to generate a general index of psychopathology. Following Allegrini et al. (2020), we modelled parent‐ and twin‐rated p‐factors separately, as they are moderately correlated but have distinct aetiologies, with parent‐rated p showing significantly larger shared environmental influences than twin‐rated p. We used factor scores derived from the CFA for subsequent analyses.

We had only two variables for twin‐rated home environment, so CFA was not feasible. Instead, we used principal component analysis (PCA), which indicated that both components had equal loading onto the first principal component. Therefore, the mean of the two variables was used as the twin‐rated home environment index at each age.

#### Measuring the association between the p‐factor and home environment

**Figure 1:**
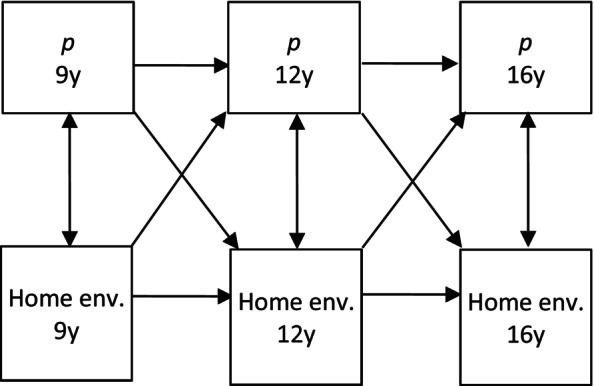
A cross‐lagged panel model of p‐factor and home environment across three time points (age 9, 12, 16)

Correlations between measures were calculated to observe levels of association. Phenotypic, genetic, and MZ differences analyses were conducted using cross‐lagged panel modelling. Cross‐lagged effects represent the relationship between two variables when their stability and associations over time are controlled (Cole & Maxwell, 2003; Malanchini et al., 2017). This method is, therefore, appropriate for estimating bidirectional, longitudinal relationships, in this case, between p‐factor and twin‐rated home environment after accounting for each measure's stability and the cross‐sectional relationship at previous time points. Models were estimated for both parent‐ and twin‐rated p‐factor at ages 9, 12, and 16. Informant ratings were kept separate given that the associations between parent‐ and twin‐rated p‐factor measures indicated low to moderate commonality across raters (*M* = 0.17 range = −0.04, 0.52]). This corroborates the frequently reported discrepancy between parent‐ and child ratings of psychopathology (e.g. De Los Reyes et al., 2011, 2012). Figure 1 shows the basic structure of the model used in the present study. We used bootstrapped 95% confidence intervals to interpret the strengths of the cross‐lagged, cross‐sectional, and stability paths.

#### Examining the aetiology of the longitudinal associations between p‐factor and home environment

We used the twin method to examine the aetiology of the p‐factor and twin‐rated home environment and the covariation between them. Twin methods capitalise on the genetic relatedness between monozygotic or identical (MZ) and dizygotic or non‐identical (DZ) twins. MZ twins share 100% of their genes, whilst DZ twins share, on average, 50% of their segregating genes. However, both sets of twins share their rearing environment. By comparing the difference between MZ and DZ correlations, it is possible to estimate the relative contribution of genetic, shared environmental, and nonshared environmental effects to individual differences in the trait of interest. Heritability (A), the proportion of variance explained by the genetic factors, can be calculated by doubling the difference between MZ and DZ twins. Shared environmental factors (C), which make children growing up in the same family similar beyond those explained by genetic factors, can be calculated by deducting heritability from the MZ correlation. The rest of the variance is explained by nonshared environmental factors (E), environmental factors that do not contribute to similarities between twins growing up in the same family, which can be calculated by deducting MZ correlations from unity, which also includes measurement error. The ACE estimates can be calculated more accurately, including 95% confidence intervals using structural equation modelling (Knopik et al., 2017; Martin & Eaves, 1977; Rijsdijk & Sham, 2002). The present study used an OpenMx package in R for all twin analyses (Boker et al., 2011).

Univariate twin analyses can be extended to bivariate genetic analyses to study the aetiology of covariance between traits. The covariance between traits can be decomposed into additive genetic (A), shared environmental (C), and nonshared environmental (E) components by comparing the cross‐twin cross‐trait correlations between MZ and DZ twin pairs (Rijsdijk & Sham, 2002). This method was used to identify the aetiology of each cross‐lagged association between the p‐factor and twin‐rated home environment. This analysis was not included in the preregistration but was later determined to be an important contribution.

The cross‐lagged ACE model, the TwinAnalysis package for R (https://github.com/IvanVoronin/TwinAnalysis), is an implementation of a multivariate twin model. It uses the cross‐trait cross‐twin correlations in MZ and DZ twin pairs to estimate the cross‐lagged relationships between the additive genetic, shared environmental, and nonshared environmental factors underlying the relationships between variables. These estimates are then used to calculate the proportion of phenotypic variance explained by A, C, and E. Such an approach, detailed by Malanchini et al. (2017) and utilised in subsequent studies (e.g. McAdams, Rijsdijk, Zavos, & Pingault, 2021), offers evidence on likely directions of influence, integrating all path analyses within a single model, facilitating direct comparisons of cross‐trait influences.

#### MZ differences design

The MZ differences design was also used to confirm the robustness of our findings by isolating nonshared environmental factors whilst controlling for genetic and shared environmental influences. This allowed us to assess whether the results align with those from the cross‐lagged model. Given that MZ twins share 100% of their genetics and shared environment, any differences between them are attributable to nonshared environmental influences (Liang & Eley, 2005; Ritchie, Bates, & Plomin, 2015). This allowed us to estimate the extent to which differences in perceptions of the home environment and the associations between these perceptions with parent‐ or self‐reported p are influenced by nonshared environmental influences—environmental factors that are individual‐specific and do not contribute to similarities between twin pairs. Focusing on within‐pair differences among MZ twins, this approach provides a test of environmental influences independent of genetic predispositions and shared environment, complementing the multivariate twin analyses.

## Results

### Phenotypic analyses

#### Descriptive statistics

Means and standard deviations were calculated for measures for the whole sample, males and females, separately; these are presented in Table S1. Analyses of variance (ANOVA) were used to test the significance of these group differences. Significant sex differences emerged for all variables, except twin‐rated home environment variables at age 16 and MFQ at age 12. The effect sizes of these sex differences were modest, with sex explaining, on average, around 1% of the variance, except 10% of the variance for SDQ emotional problems at age 16, which is in line with prior research (e.g., Eme, 1979; Rutter, 1988). The average correlation between variables and age when they were collected was .02, ranging from .01 to .05. Measures were subsequently corrected via multiple regressions for sex and age. These age and sex‐corrected standardised residuals were used in all downstream analyses.

#### Correlations

As expected, the psychopathology measures were moderately correlated amongst their respective raters across ages. Figures S1–S3 present the correlation heatmaps for all adjusted phenotypic variables at waves 9, 12, and 16, respectively, including correlations between phenotypic measures for single informants and twin‐rated home environment measures.

Figure S4 shows the correlations between the p‐factor and twin‐rated home environment composites at each age and by each rater. Parent‐rated p‐factors correlated positively over time (*r* = .53–.71), whereas twin‐rated p‐factor correlations ranged from weak to moderate (*r* = .21–.46). P‐factor correlations between raters were also moderate (*r* = .23–.46). Twin‐rated home environment factors were also moderately positively correlated with each other (*r* = .25–.44) and also correlated with parent‐rated p‐factors (*r* = .18–.30, *M* = 0.18) and twin‐rated p‐factors (*r* = .14–.40).

#### Computing latent factors of mental health (p‐factor)

We calculated a p‐factor, that is, a general factor for psychopathology for each age group, using confirmatory factor analyses (CFA). Here, we were not interested in the structure of mental health problems across childhood but used CFA as a data reduction technique to derive p‐factors across raters to index mental health problems at every age. All psychopathology measures loaded onto the twin‐rated p‐factor at all ages. The same applies to the parent‐rated p‐factor except for the AQ Attention to Detail scale at age 16. The factor loadings and model‐fit statistics are presented in Table S2, and model‐fit statistics are presented in Table S3.

#### Cross‐lagged analyses

##### Parent‐rated p‐factor model

**Figure 2:**
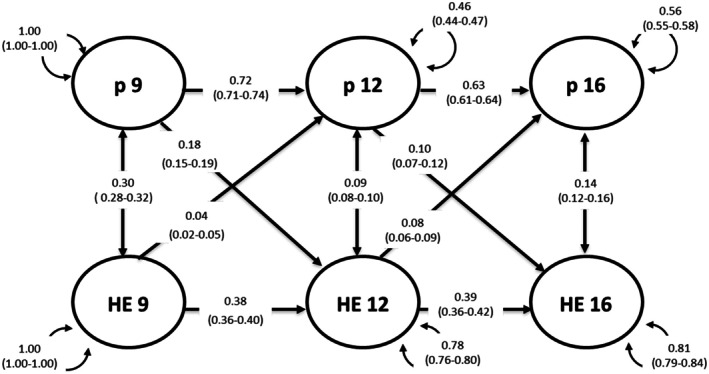
A cross‐lagged panel model between the parent‐rated p‐factor and twin‐rated home environment composite. Standardised path estimates, 95% CI, are in brackets. Solid lines indicate statistically significant path estimates. HE, twin‐rated home environment, P, parent‐reported p‐factor

All autoregressive, cross‐sectional, and cross‐lagged paths were statistically significant, as presented in Figure 2. Both latent factors demonstrated stability over time with less variability in p‐factor compared with twin‐rated home environment. After controlling for the stability of p‐factor and twin‐rated home environment and their cross‐sectional correlations, the cross‐lagged paths were also significant. Although the effect sizes were small, on average the p‐factor had a larger effect on twin‐rated home environment at both time points (9–12, *β* = .18 (95% CI: 0.15–0.19); 12–16, *β* = .10 (95% CI: 0.07–0.12)) than the reverse (9–12, *β* = .04 (95% CI: 0.02–0.05); 12–16, *β* = .08 (95% CI: 0.06–0.09)). Model‐fit statistics for cross‐lagged models are presented in Table S4.

##### Twin‐rated p‐factor model

**Figure 3:**
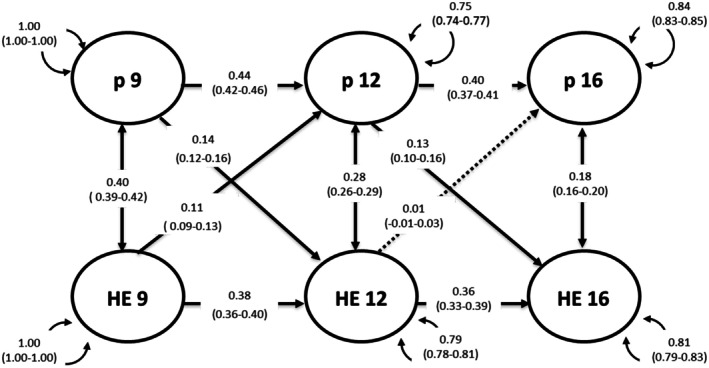
A cross‐lagged panel model between the twin‐rated p‐factor (p) and twin‐rated home environment composite. Standardised path estimates, 95% CI, are in brackets. Solid lines indicate statistically significant path estimates. HE, twin‐rated home environment; P, twin‐rated p‐factor

Except for the pathway home environment at 12 and p‐factor at 16, all autoregressive, cross‐sectional, and cross‐lagged paths were again statistically significant, as illustrated in Figure 3. Both latent factors demonstrated stability over time with less variability in p‐factor compared with twin‐rated home environment. There was a bidirectional relationship between p‐factor at 9 and twin‐rated home environment at 12 (*β* = .14 (95% CI: 0.12–0.16)) and from twin‐rated home environment at 9 to p‐factor at 12 (*β* = .11 (95% CI: 0.09–0.13)) after controlling for the stability of both constructs and their cross‐sectional correlations. However, there was only a unidirectional relationship from p‐factor at 12 to twin‐rated home environment at 16 (*β* = .13 (95% CI: 0.10–0.16)). Model‐fit statistics for cross‐lagged models are presented in Table S4.

### Genetic analyses

#### ACE cross‐lagged analyses

According to the fit statistics, all models provided an accurate description of the data (Table S4).

#### Parent‐rated p‐factor ACE model

The twin correlations and cross‐twin cross‐trait correlations are presented in Table S5. The heritability of parent‐rated p‐factor was consistently high (age 9 – *h* ^2^ = 0.51 [0.47–0.58]; age 12 – *h* ^2^ = 0.67 [0.63–0.71]; age 16 – *h* ^2^ = 0.59 [0.55–0.64]), whereas the heritability of twin‐rated home environment was consistently lower (age 9 – *h* ^2^ = 0.19 [0.13–0.27]; age 12 – *h* ^2^ = 0.19 [0.12–0.24]; age 16 – *h* ^2^ = 0.31 [0.21–0.41]). The remaining variance was accounted for by shared and nonshared environmental factors (see Tables S6 and S7).

**Figure 4:**
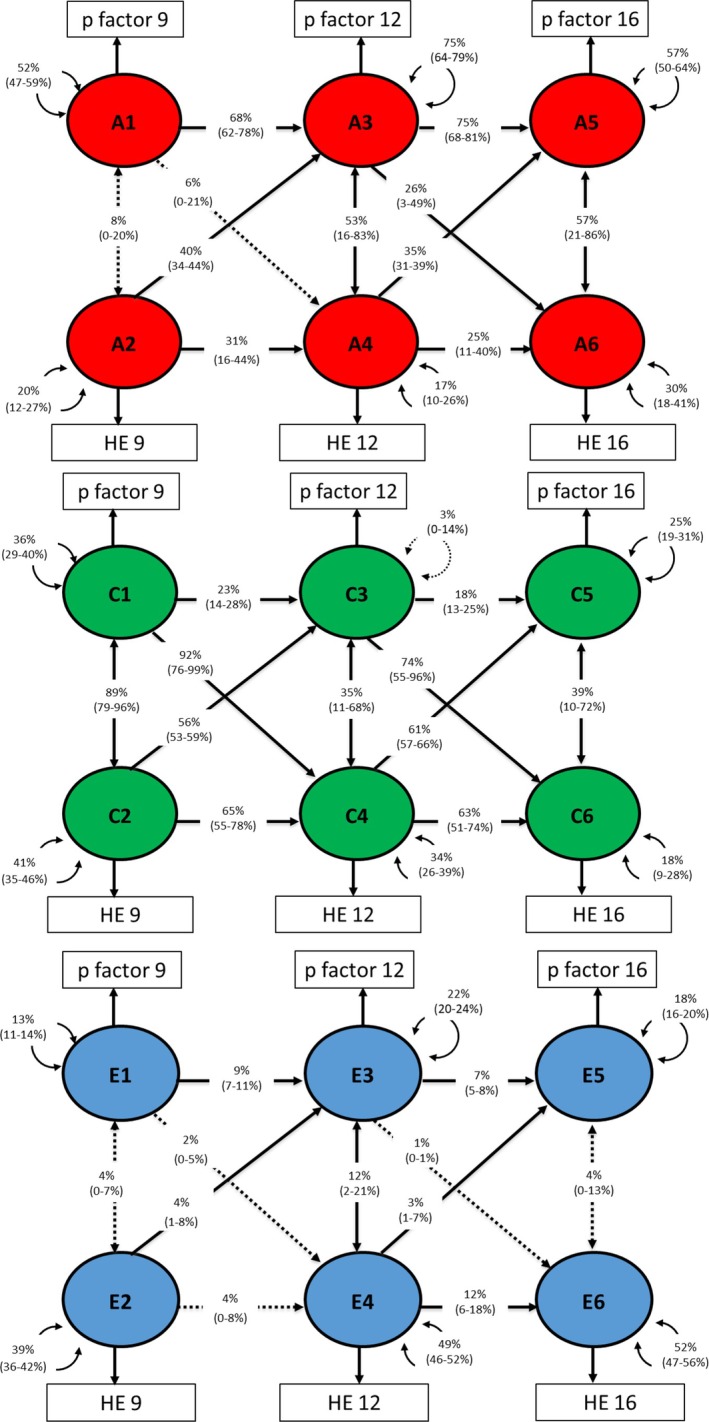
Genetic and environmental decomposition of associations between parent‐rated p‐factor and twin‐rated home environment at ages 9, 12, and 16. ‘A’ represents the proportion of variance (%) explained by additive genetic effects, ‘C’ by shared environment and ‘E’ by nonshared environment

Genetic and environmental decomposition of the developmental associations between parent‐rated p‐factor and twin‐rated home environment is presented in Figure 4. The moderate stability of the p‐factor was mostly explained by A (68% from age 9–12 and 75% from 12 to 16). Twin‐rated home environment stability was mostly explained by C (65% from age 9–12 and 63% from age 12–16). Cross‐lagged pathways from p‐factor to twin‐rated home environment were also largely explained by shared environmental factors, with a smaller proportion explained by genetic factors.

#### Twin‐rated p‐factor ACE model

The twin correlations and cross‐twin cross‐trait correlations are presented in Table S5. The heritability of twin‐rated p‐factor was moderate (age 9 – *h* ^2^ = 0.47 [0.41–0.51]; age 12 – *h* ^2^ = 0.42 [0.37–0.47]; age 16 – *h* ^2^ = 0.46 [0.41–0.51]). The remaining variance was accounted for by shared and nonshared environmental factors (see Tables S8 and S9).

**Figure 5:**
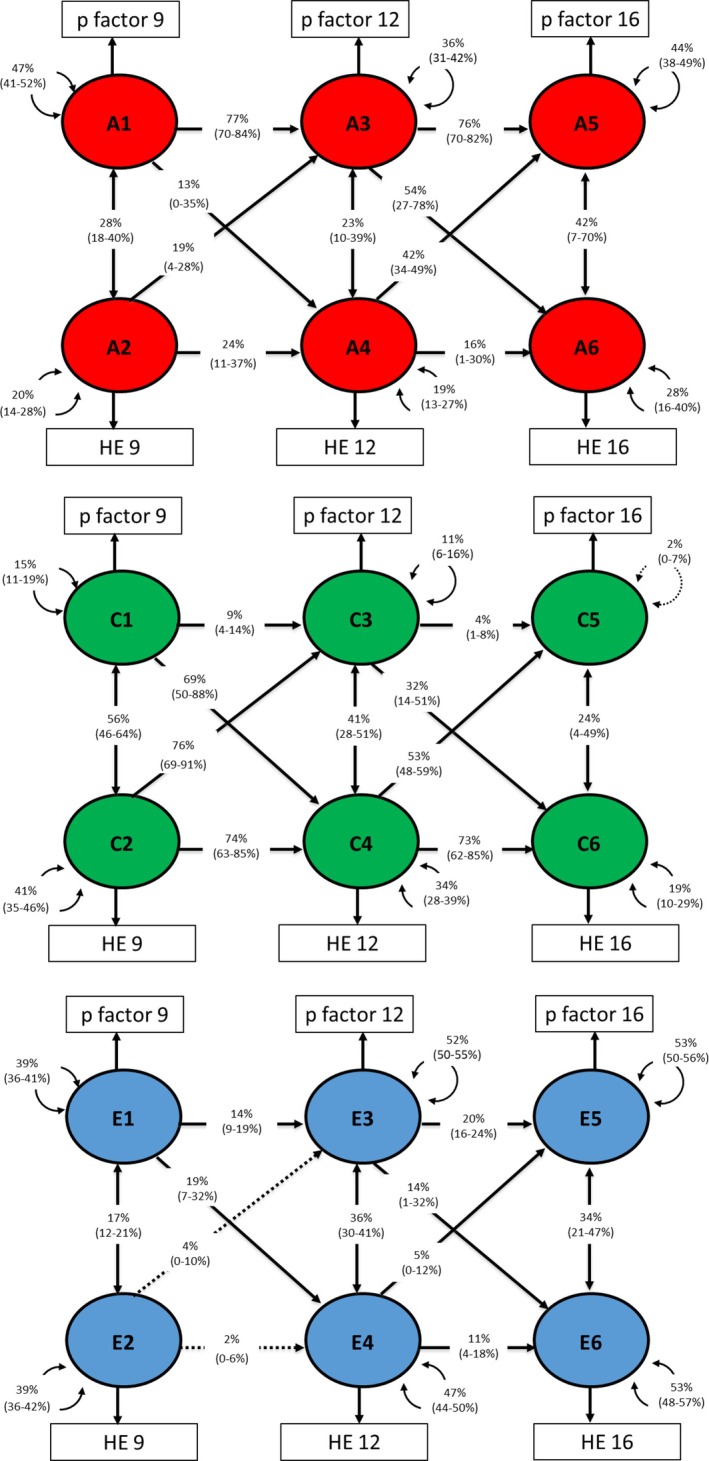
Genetic and environmental decomposition of associations between twin‐rated p‐factor and twin‐rated home environment at ages 9, 12, and 16. ‘A’ represents the proportion of variance (%) explained by additive genetic effects, ‘C’ by shared environment and ‘E’ by nonshared environment

Genetic and environmental decomposition of the developmental associations between twin‐rated p‐factor and twin‐rated home environment is presented in Figure 5. The moderate stability of p‐factor was mostly explained by A (77% from age 9 to 12 and 76% from 12 to 16). Home environment stability was mostly explained by C (74% from age 9 to 12 and 73% from age 12 to 16). Cross‐lagged pathways from p‐factor to the home environment were also largely explained by shared environmental factors, except the cross‐lagged path from p‐factor at 12 to the home environment at 16, which was 54% explained by genetic factors, 32% by shared environmental factors, and 14% by nonshared environmental factors.

We also conducted separate analyses for twin‐rated home chaos and parental discipline. Univariate genetic results are presented in Table S10. Phenotypic cross‐lagged models between p and home chaos are presented in Figures S5 and S7, and in Figures S9 and S11 between p and parental discipline. Genetic models between p and home chaos are presented in Figures S6 and S8, and in Figures S10 and S12 between p and parental discipline. The results were largely similar, further supporting the robustness of our findings. Model‐fit statistics are presented in Table S4.

### MZ differences analyses

#### Parent‐rated p‐factor model

MZ twin difference scores were calculated by subtracting Twin 2's score from Twin 1's score for each variable, randomly assigning twin order. This method captures within‐pair differences whilst controlling for genetic and shared environmental influences. MZ twin difference scores were small on average but exhibited substantial variability (e.g. twin‐rated p at age 9 mean = 0.004, *SD* = 0.876). These difference scores were approximately normally distributed, as given in Table S11. Correlations between parental ratings and twin‐rated home environment differences were small, averaging 0.07. The stability of these scores was also low, with an average correlation of 0.24, and the highest stability was for parent‐rated p, which was 0.38. Refer to Table S12 for details. No significant cross‐lagged paths were found between parent‐rated p‐factor and twin‐rated home environment when controlling for genetics and shared environment (see Figure S13). The p‐factor remained stable over time (mean *β* = .34–.45), whereas twin‐rated home environment only demonstrated stability from age 12–16 (*β* = .08). Cross‐sectional paths were not statistically significant. The attenuation of the associations, compared with the regular phenotypic cross‐lagged model, confirms that the stability and cross‐lagged relationships between twin‐rated home environment and p‐factors are attributed mostly to the genetic and shared environmental effects.

#### Twin‐rated p‐factor model

Except for the pathway from p‐factor at 9 to the home environment at 12 (*β* = .09), no significant cross‐lagged paths were found between the twin‐rated p‐factor and the home environment (see Figure S14). As before, p‐factor remained stable over time (mean *β* = .16–.19), whereas the home environment only demonstrated stability from age 12–16 (*β* = .08), but estimates were small in both cases.

We also conducted analyses separately for chaos and discipline; the results are in line with results shown for home environment composite. The only significant cross‐lagged path is from twin‐rated p at 9 to twin‐rated discipline at 12 (*β* = .07; See Figures S15–S18).

## Discussion

Using a cross‐lagged panel model, our study explored the longitudinal associations between twin‐rated home environment and general psychopathology (p) factor during key developmental phases. The results illustrate that the perceptions of the household environment, specifically chaos and parental discipline, are predictive of later psychopathological outcomes. Conversely, early indicators of the p‐factor can forecast subsequent household conditions. Notably, the reciprocal relationships between these domains were observed independently of their concurrent intercorrelations and stability. Although the effects identified were modest, our results suggest that interventions to improve household dynamics might also positively impact psychopathological trajectories.

Our study's robustness stems from genetically informed designs, including genetic cross‐lagged models and an MZ differences design. This approach revealed that the stability of the p‐factor over time was mainly due to genetic influences, whilst the shared environmental factors mainly explained the relative stability of the twin‐rated home environment. In contrast, shared environmental factors predominantly shaped the bidirectional influences between these two factors.

Whilst the p‐factor is known to be heritable, its interplay with the home environment over time appears to involve both genetic and environmental factors. Shared environmental influences play a particularly important role in explaining the associations between the home environment and mental health, suggesting that these links are shaped by experiences common to twins rather than genetic differences. However, genetic factors also contribute to individual differences in how the home environment is perceived, illustrating gene–environment correlation. Furthermore, genetic influences explain the association between the p‐factor and the home environment, though to a lesser degree than shared environmental factors—again highlighting gene–environment interplay. This likely reflects both passive and evocative gene–environment correlation, where genetically influenced traits shape the environments individuals encounter and the responses they elicit.

The MZ differences design provides an additional layer of insight by accounting for both genetic and shared environmental influences. By examining within‐twin pair differences, this approach isolates the role of nonshared environmental influences in shaping the interplay between twin‐rated home environment and the p‐factor. The absence of significant cross‐lagged associations in this design suggests that nonshared environmental influences do not primarily drive these bidirectional relationships but instead reflect genetic and shared environmental contributions. The exception to this was twin‐rated p at age 9, which influenced how twins perceived the home environment, as well as parental discipline, although the effect was small and not significant at other ages.

Despite modest correlations between parent and twin ratings of p‐factor, the findings were remarkably consistent across both models. Both demonstrated high heritability for p‐factor and moderate stability over time, primarily driven by genetic factors. The twin‐rated model showed less stability in p‐factor compared with the parent‐rated model. Shared environmental factors largely explained the cross‐lagged pathways in both models, but genetic factors contributed more significantly to the twin‐rated model, especially for the path from the p‐factor at age 12 to the home environment at age 16. This suggests that whilst genetic factors underpin the stability of p‐factor, the interplay between twin‐rated home environment and psychopathology is shaped more by environmental experiences that are shared within families. The nonshared environmental component explained more variance in twin‐rated p (~40%), reflecting child‐specific effects. However, even in parent‐rated p, where rater bias is reduced, shared environmental factors predominantly explained the cross‐lagged effects between p and twin‐rated home environment. Small but significant nonshared environmental effects were observed from p to home environment in the twin‐rated model, but not in the parent‐rated model, and from home environment to parent‐rated p‐factor but not to twin‐rated p. This suggests that whilst genetic factors underpin the stability of p‐factor, the interplay between home environment and psychopathology is shaped more by environmental experiences that are shared within families. Whilst we fully acknowledge that these models do not provide definitive causal proof and that unmeasured confounding cannot be ruled out, our interpretation rests on the consistency of the reciprocal associations over time and across informants, rather than on any single parameter alone.

The current findings reiterate the importance of multiinformant assessment. The analyses revealed that twin‐rated home environment at age 12 is associated with p‐factor at 16 in the parent‐rated model but not in the twin‐rated model. The use of age‐appropriate measures for young people across development, alongside valid parental measures for the same constructs, remains key. Although effect sizes were small, these findings suggest potential intervention targets at both individual and family levels. Providing psychoeducation on how certain child traits may contribute to perceptions of household chaos and parental discipline, alongside strategies for managing environmental stressors, could be one approach. Moreover, the likely genetic influence found here may indicate that family‐based transdiagnostic treatment can yield collective positive outcomes, perhaps by focusing on shared aetiological factors between family members. Establishing standardised assessments of the home environment and genetically influenced traits such as caregiver psychopathology may flag appropriate families for whom this approach is most beneficial. Future research should consider expanding measures of the home environment beyond chaos and discipline to capture a broader range of experiences that may contribute to shared family influences on psychopathology.

The general factor of psychopathology, ‘p’, represents commonalities across mental disorders, derived statistically from symptom correlations. It is a statistical construct, and there is no consensus on what p‐factor really is, which limits the present study's findings (see Lane, Steinley, & Sher, 2016; Watts, Lane, Bonifay, Steinley, & Meyer, 2020). More widely, a consensus on measuring p‐factor is needed (Fried, Greene, & Eaton, 2021; Lahey, Moore, Kaczkurkin, & Zald, 2021). This may come via dimensional rather than diagnostic measures or norms weighted by their loadings onto p‐factor (Pettersson, Lahey, Larsson, & Lichtenstein, 2018). Treatment modality may also be discernible where individuals with broad comorbidity score highly on specific factors after isolating p‐factor (Pettersson, Larsson, & Lichtenstein, 2021). The present study attempted to overcome this by replicating the hypothesis‐free approach of a previous same‐sample study (Allegrini et al., 2020). The primary aim was to investigate the longitudinal interplay between general psychopathology and twin‐rated home environment, not to verify the psychopathology's underlying factor structure.

### Limitations and future directions

There are several limitations to consider. The primary limitation of our study lies in the diversity of measurement instruments used to assess p‐factor across different assessment waves. This variation prompts questions about whether observed developmental changes are due to actual shifts in the underlying constructs or differences in measurement tools. Despite using age‐appropriate and valid measures, further research is necessary to ensure these tools consistently capture the same constructs over time (Von Stumm et al., 2024).

Although we used the same home environment measures developmentally, we were limited to twin‐rated household chaos and parental discipline only, as these were only two measures consistently collected at each age wave pertaining to this construct. The reliance on self‐reported data for assessing the home environment could introduce biases that do not fully reflect the actual dynamics within the household. A broader measure of the home environment could also account for the proximity and severity of exposure to neighbourhood disorder, housing conditions, and relationship instability, all of which are indicative of future psychopathology (Coley, Lynch, & Kull, 2015). Future studies might benefit from incorporating objective measurements or observational data for a more comprehensive analysis.

Parent‐reported perceptions of home environment measures were omitted here but could add to clinical understanding and provide objective and subjective information to assess the overall environment. Measures such as the Home Observation for Measurement of the Environment Scale (HOME; Caldwell & Bradley, 2003) may better operationalise this construct by including physical and relational aspects (e.g. cleanliness, safety, parental responsiveness).

Another consideration is the timing of our data collection. Since assessments were conducted every few years, important nuances in the developmental trajectory of the p‐factor concerning changes in the home environment might have been overlooked. More frequent data collection could help elucidate these dynamics more clearly. Another limitation is a lack of direct measures of pubertal timing and progression. Adolescence is a time of change: shifts in hormones and the body, changes in the social environment, and modifications in the brain and mind. Although most young people develop into healthy adults, adolescence brings vulnerabilities to mental health issues (Blakemore, 2019), which may also impact perceptions of the home environment.

Our findings' generalisability may also be limited due to our study's specific demographic and geographic focus. Extending this research to a broader range of populations would help to assess how well the findings generalise to diverse populations.

Lastly, it is argued that when stable traits such as p‐factor are modelled using cross‐lagged panel modelling, cross‐lagged relationships are confounded by between‐person differences, meaning within‐person relationships are more challenging to infer causally (Hamaker, Kuiper, & Grasman, 2015). An alternative methodological approach, random‐intercept cross‐lagged panel modelling (RI‐CLPM), may be more suitable, having already supported dimensional comorbidity in 7–12‐year‐olds and their families (Allegrini et al., 2022). However, this study's nonshared environmental component and MZ difference scores also account for time‐constant confounds, addressing concerns similar to those of RI‐CLPM. Given our focus on the genetic and environmental contributions to longitudinal associations, CLPM was the more appropriate choice for this study.

## Conclusion

The present study aimed to build on the emerging p‐factor literature by investigating its longitudinal association with twin‐rated home environment. Whilst the study points to a significant bidirectional relationship influenced by both genetic and environmental elements, it is essential to interpret these findings within the context of current methodological limitations. Our findings suggest that whilst genetic influences contribute to the stability of psychopathology, the interplay between home environment and p‐factor is shaped more by shared environmental experiences, particularly household chaos and parental discipline. This aligns with developmental frameworks that emphasise the role of environmental continuity in shaping mental health trajectories (Marsh, Dobson, & Maddison, 2020).

The complexity and variability inherent in measuring the ‘p‐factor’ and the home environment caution against drawing definitive conclusions about their developmental interplay. Perhaps, more evident are age‐specific processes, which create a complex interplay between genes and the environment throughout development. Although the effect sizes between home environment and psychopathology were modest, the results suggest that interventions that enhance consistency in parental discipline and reduce chaotic home environments may help alter trajectories of general psychopathology. These preliminary insights, though valuable, underscore the need for further, more detailed research to clarify these relationships and their implications for childhood psychopathology. Operationalising the relevant indicators of p‐factor and home environment will improve understanding of these processes and benefit clinical practice by informing family‐based interventions. Future research should incorporate more frequent assessments to track short‐term variations in home environment and psychopathology, whilst also examining broader contextual influences beyond the household, including peer relationships and school environments. We hope this study serves as a stepping stone towards a deeper understanding rather than a conclusive guide for clinical application.

## Ethical considerations

Written informed consent was obtained from parents before data collection and from TEDS participants themselves past the age of 18. King's College London's Ethics Committee approved the project in March 2020 for the Institute of Psychiatry, Psychology and Neuroscience PNM/09/10‐104.

**Key points**

**What's known?**

The relationship between the p‐factor and twin‐rated home environment is dynamic and bidirectional, indicating that changes in one can influence the other across different developmental stages. However, the effect sizes of these relationships were modest.

**What's new?**

Shared environmental factors played a major role in driving cross‐lagged associations between the p‐factor and twin‐rated home environment, with some genetic contribution. This suggests that the family environment can significantly shape this relationship.

**What's relevant?**

These findings call for further investigation into the gene–environment interplay in shaping psychopathology. A better understanding of these dynamics could inform effective prevention and intervention strategies for developmental psychopathology.

## Supporting information


**Table S1.** Descriptive statistics for phenotypic variables and ANOVA results testing for sex differences (using raw scores for phenotypic analysis sample only).
**Table S2.** Factor loadings for the p factor model across ages and raters.
**Table S3.** Model fit statistics for the p factor model across ages and raters.
**Table S4.** Model fit statistics for phenotypic and genetic cross‐lagged panel models.
**Table S5.** Cross‐twin and cross‐trait correlations.
**Table S6.** Twin model‐fitting results for univariate analyses parent‐rated p factor and home environment.
**Table S7.** Percentages of genetic and environmental variance unique to each construct in parent‐rated model after accounting for variance shared with previous time points.
**Table S8.** Twin model‐fitting results for univariate analyses of twin‐rated p factor and home environment.
**Table S9.** Percentages of genetic and environmental variance unique to each construct in twin‐rated model after accounting for variance shared with previous time points.
**Table S10.** Twin model‐fitting results for univariate analyses of parent‐ and twin‐rated p factor, twin‐rated CHAOS and twin‐rated parental discipline.
**Table S11.** Descriptive statistics for the MZ difference scores.
**Table S12.** A correlation matrix for MZ difference scores.
**Table S1.** Descriptive statistics for phenotypic variables and ANOVA results testing for sex differences (using raw scores for phenotypic analysis sample only).
**Table S2.** Factor loadings for the p factor model across ages and raters.
**Table S3.** Model fit statistics for the p factor model across ages and raters.
**Table S4.** Model fit statistics for phenotypic and genetic cross‐lagged panel models.
**Table S5.** Cross‐twin and cross‐trait correlations.
**Table S6.** Twin model‐fitting results for univariate analyses parent‐rated p factor and home environment.
**Table S7.** Percentages of genetic and environmental variance unique to each construct in parent‐rated model after accounting for variance shared with previous time points.
**Table S8.** Twin model‐fitting results for univariate analyses of twin‐rated p factor and home environment.
**Table S9.** Percentages of genetic and environmental variance unique to each construct in twin‐rated model after accounting for variance shared with previous time points.
**Table S10.** Twin model‐fitting results for univariate analyses of parent‐ and twin‐rated p factor, twin‐rated CHAOS and twin‐rated parental discipline.
**Table S11.** Descriptive statistics for the MZ difference scores.
**Table S12.** A correlation matrix for MZ difference scores.
**Figure S1.** A correlation heatmap for all phenotypic variables at age 9.
**Figure S2.** A correlation heatmap for all phenotypic variables at age 12.
**Figure S3.** A correlation heatmap for all phenotypic variables at age 16.
**Figure S4.** A correlation heatmap for all composites.
**Figure S5.** A phenotypic cross‐lagged panel model between the parent‐rated p factor and twin‐rated CHAOS.
**Figure S6.** Genetic and environmental decomposition of associations between parent‐rated p factor and twin‐rated CHAOS at ages 9, 12 and 16.
**Figure S7.** A phenotypic cross‐lagged panel model between the twin‐rated p factor and twin‐rated CHAOS.
**Figure S8.** Genetic and environmental decomposition of associations between twin‐rated p factor and twin‐rated CHAOS at ages 9, 12 and 16.
**Figure S9.** A phenotypic cross‐lagged panel model between the parent‐rated p factor and twin‐rated parental discipline.
**Figure S10.** Genetic and environmental decomposition of associations between parent‐rated p factor and twin‐rated ‘Parental Discipline’ at ages 9, 12 and 16.
**Figure S11.** A phenotypic cross‐lagged panel model between the twin‐rated p factor and twin‐rated parental discipline.
**Figure S12.** Genetic and environmental decomposition of associations between twin‐rated p factor and twin‐rated ‘Parental Discipline’ at ages 9, 12 and 16.
**Figure S13.** A cross‐lagged panel model between the parent‐rated p factor (p) and home environment composite (HE) MZ difference scores.
**Figure S14.** A cross‐lagged panel model between the twin‐rated p factor (p) and home environment composite (HE) MZ difference scores.
**Figure S15.** A cross‐lagged panel model between the parent‐rated p factor (p) and twin‐rated CHAOS MZ difference scores.
**Figure S16.** A cross‐lagged panel model between the twin‐rated p factor (p) and twin‐rated CHAOS MZ difference scores.
**Figure S17.** A cross‐lagged panel model between the parent‐rated p factor (p) and twin‐rated parental discipline MZ difference scores.
**Figure S18.** A cross‐lagged panel model between the twin‐rated p factor (p) and twin‐rated parental discipline MZ difference scores.

## Data Availability

For information on data availability, please see the TEDS data access policy. This can be found at: http://www.teds.ac.uk/research/collaborators‐and‐data/teds‐data‐access‐policy. All relevant data are available from the authors according to the TEDS data access policy.
