# Supplementary material for: How are children's perceptions of the home environment associated with a general psychopathology factor across childhood?
Source: J Child Psychol Psychiatry. 2025 Sep 8;67(2):266–81. doi: 10.1111/jcpp.70046 (PMC12812785; doi:10.1111/jcpp.70046)
Supplement: Supplementary file 1 — Table S1. Descriptive statistics for phenotypic variables and ANOVA results testing for sex differences (using raw scores for phenotypic analysis sample only). Table S2. Factor loadings for the p factor model across ages and raters. Table S3. Model fit statistics for the p factor model across ages and raters. Table S4. Model fit statistics for phenotypic and genetic cross‐lagged panel models. Table S5. Cross‐twin and cross‐trait correlations. Table S6. Twin model‐fitting results for univariate analyses parent‐rated p factor and home environment. Table S7. Percentages of genetic and environmental variance unique to each construct in parent‐rated model after accounting for variance shared with previous time points. Table S8. Twin model‐fitting results for univariate analyses of twin‐rated p factor and home environment. Table S9. Percentages of genetic and environmental variance unique to each construct in twin‐rated model after accounting for variance shared with previous time points. Table S10. Twin model‐fitting results for univariate analyses of parent‐ and twin‐rated p factor, twin‐rated CHAOS and twin‐rated parental discipline. Table S11. Descriptive statistics for the MZ difference scores. Table S12. A correlation matrix for MZ difference scores. Table S1. Descriptive statistics for phenotypic variables and ANOVA results testing for sex differences (using raw scores for phenotypic analysis sample only). Table S2. Factor loadings for the p factor model across ages and raters. Table S3. Model fit statistics for the p factor model across ages and raters. Table S4. Model fit statistics for phenotypic and genetic cross‐lagged panel models. Table S5. Cross‐twin and cross‐trait correlations. Table S6. Twin model‐fitting results for univariate analyses parent‐rated p factor and home environment. Table S7. Percentages of genetic and environmental variance unique to each construct in parent‐rated model after accounting for variance shared with previous time points. Table [file JCPP-67-266-s001.docx]

**Supplementary Material**

**How are children’s perceptions of the home environment associated with a general psychopathology factor across childhood?**

**Jack K. Nejand, Margherita Malanchini, Ivan Voronin, Thalia C. Eley and Kaili Rimfeld**

Table of Contents

[Supplementary Tables 3](#_Toc202523438)

[Table S1. *Descriptive statistics for phenotypic variables and ANOVA results testing for sex differences (using raw scores for phenotypic analysis sample only)* 3](#_Toc202523439)

[Table S2. *Factor loadings for the p factor model across ages and raters.* 6](#_Toc202523440)

[Table S3. *Model fit statistics for the p factor model across ages and raters.* 8](#_Toc202523441)

[Table S4. *Model fit statistics for phenotypic and genetic cross-lagged panel models* 9](#_Toc202523442)

[Table S5. *Cross-twin and cross-trait correlations.* 10](#_Toc202523443)

[Table S6. *Twin model-fitting results for univariate analyses parent-rated p factor and home environment. A = additive genetic, C = shared environmental, E = non-shared environmental proportions of the variance.* 14](#_Toc202523444)

[Table S7. *Percentages of genetic and environmental variance unique to each construct in parent-rated model after accounting for variance shared with previous time points. A = additive genetic, C = shared environmental, E = non-shared environmental proportions of the variance.* 14](#_Toc202523445)

[Table S8. *Twin model-fitting results for univariate analyses of twin-rated p factor and home environment. A = additive genetic, C = shared environmental, E = non-shared environmental proportions of the variance.* 15](#_Toc202523446)

[Table S9. *Percentages of genetic and environmental variance unique to each construct in twin-rated model after accounting for variance shared with previous time points. A = additive genetic, C = shared environmental, E = non-shared environmental proportions of the variance.* 15](#_Toc202523447)

[Table S10. *Twin model-fitting results for univariate analyses of parent- and twin-rated p factor, twin-rated CHAOS and twin-rated parental discipline. A = additive genetic, C = shared environmental, E = non-shared environmental proportions of the variance.* 16](#_Toc202523448)

[Table S11. *Descriptive statistics for the MZ difference scores* 17](#_Toc202523449)

[Table S12. *A correlation matrix for MZ difference scores*. 18](#_Toc202523450)

[Supplementary Figures 19](#_Toc202523451)

[Figure S1. *A correlation heatmap for all phenotypic variables at age 9* 19](#_Toc202523452)

[Figure S2. *A correlation heatmap for all phenotypic variables at age 12* 20](#_Toc202523453)

[Figure S3. *A correlation heatmap for all phenotypic variables at age 16* 21](#_Toc202523454)

[Figure S4. *A correlation heatmap for all composites* 22](#_Toc202523455)

[Figure S5. *A phenotypic cross-lagged panel model between the parent-rated p factor and twin-rated CHAOS.* 23](#_Toc202523456)

[Figure S6. Genetic and environmental decomposition of associations between parent-rated p factor and twin-rated CHAOS at ages 9, 12 and 16. ‘A’ represents the proportion of variance (%) explained by additive genetic effects, ‘C’ by shared environment and ‘E’ by non-shared environment*.* 24](#_Toc202523457)

[Figure S7. *A phenotypic cross-lagged panel model between the twin-rated p factor and twin-rated CHAOS.* 27](#_Toc202523458)

[Figure S8. Genetic and environmental decomposition of associations between twin-rated p factor and twin-rated CHAOS at ages 9, 12 and 16. ‘A’ represents the proportion of variance (%) explained by additive genetic effects, ‘C’ by shared environment and ‘E’ by non-shared environment*.* 28](#_Toc202523459)

[30](#_Toc202523460)

[Figure S9. *A phenotypic cross-lagged panel model between the parent-rated p factor and twin-rated parental discipline.* 31](#_Toc202523461)

[Figure S10. *Genetic and environmental decomposition of associations between parent-rated p factor and twin-rated ‘Parental Discipline’ at ages 9, 12 and 16. ‘A’ represents the proportion of variance (%) explained by additive genetic effects, ‘C’ by shared environment and ‘E’ by non-shared environment.* 32](#_Toc202523462)

[Figure S11. *A phenotypic cross-lagged panel model between the twin-rated p factor and twin-rated parental discipline.* 35](#_Toc202523463)

[Figure S12. *Genetic and environmental decomposition of associations between twin-rated p factor and twin-rated ‘Parental Discipline’ at ages 9, 12 and 16. ‘A’ represents the proportion of variance (%) explained by additive genetic effects, ‘C’ by shared environment and ‘E’ by non-shared environment.* 36](#_Toc202523464)

[Figure S13. *A cross-lagged panel model between the parent-rated p factor (p) and home environment composite (he) MZ difference scores* 39](#_Toc202523465)

[Figure S14. *A cross-lagged panel model between the twin-rated p factor (p) and home environment composite (he) MZ difference scores* 40](#_Toc202523466)

[Figure S15. *A cross-lagged panel model between the parent-rated p factor (p) and twin-rated CHAOS MZ difference scores* 41](#_Toc202523467)

[Figure S16. *A cross-lagged panel model between the twin-rated p factor (p) and twin-rated CHAOS MZ difference scores* 42](#_Toc202523468)

[Figure S17. *A cross-lagged panel model between the parent-rated p factor (p) and twin-rated parental discipline MZ difference scores* 43](#_Toc202523469)

[Figure S18. *A cross-lagged panel model between the twin-rated p factor (p) and twin-rated parental discipline MZ difference scores* 44](#_Toc202523470)

|  | **Supplementary Tables****Table S1.** *Descriptive statistics for phenotypic variables and ANOVA results testing for sex differences (using raw scores for phenotypic analysis sample only)* | | | | | | | | | | | | | | |
| --- | --- | --- | --- | --- | --- | --- | --- | --- | --- | --- | --- | --- | --- | --- | --- |
| **Wave age** | | **Informant** | **Composite** | **Measure** | ***n*** | | | | | **Mean (SD)** | | | ***F value*** | ***p*** | ***R^2^*** |
|  |  |  |  |  | **Overall** | | **Male** | **Female** | | **Overall** | **Male** | **Female** |  |  |  |
| **9** | | Parent | P factor | SDQ (Prosocial) | 3407 | 1787 | | | 1620 | 8.28 (1.69) | 8.60 (1.52) | 7.93 (1.79) | 139.28 | <0.01 | 0.04 |
|  |  |  |  | SDQ (Hyperactivity) | 3403 | 1786 | | | 1619 | 3.28 (2.40) | 2.85 (2.18) | 3.74 (2.53) | 32.78 | <0.01 | 0.01 |
|  |  |  |  | SDQ (Conduct) | 3405 | 1786 | | | 1619 | 1.28 (1.44) | 1.15 (1.34) | 1.43 (1.53) | 25.28 | <0.01 | 0.01 |
|  |  |  |  | SDQ (Peer Problems) | 3405 | 1787 | | | 1618 | 1.08 (1.56) | 0.96 (1.40) | 1.22 (1.72) | 20.67 | <0.01 | 0.01 |
|  |  |  |  | SDQ (Anxiety) | 3405 | 1785 | | | 1618 | 1.78 (1.94) | 1.92 (1.96) | 1.62 (1.90) | 120.40 | <0.01 | 0.03 |
|  |  |  |  | APSD (Callous-Unemotional) | 3410 | 1786 | | | 1618 | 3.39 (1.86) | 3.12 (1.81) | 3.70 (1.86) | 36.86 | <0.01 | 0.01 |
|  |  |  |  | APSD (Narcissism) | 3404 | 1783 | | | 1615 | 1.88 (2.02) | 1.73 (1.92) | 2.05 (2.11) | 52.39 | <0.01 | 0.02 |
|  |  |  |  | APSD (Impulsivity) | 3409 | 1786 | | | 1619 | 3.29 (2.08) | 2.93 (1.99) | 3.68 (2.10) | 94.86 | <0.01 | 0.03 |
|  |  |  |  | RPAQ (Proactive Aggression) | 3404 | 1785 | | | 1616 | 0.37 (0.74) | 0.30 (0.68) | 0.45 (0.79) | 73.15 | <0.01 | 0.02 |
|  |  |  |  | RPAQ (Reactive Aggression) | 3398 | 1786 | | | 1617 | 2.01 (1.39) | 1.85 (1.35) | 2.19 (1.41) | 40.07 | <0.01 | 0.01 |
|  |  |  |  | CAST (Social) | 3405 | 1788 | | | 1622 | 2.43 (1.84) | 2.15 (1.70) | 2.75 (1.94) | 84.55 | <0.01 | 0.02 |
|  |  |  |  | CAST (Non-Social) | 3401 | 1786 | | | 1618 | 2.15 (1.57) | 1.93 (1.41) | 2.39 (1.70) | 20.98 | <0.01 | 0.01 |
|  |  |  |  | CAST (Communication) | 3403 | 1787 | | | 1622 | 2.38 (2.01) | 2.18 (1.84) | 2.61 (2.16) | 116.01 | <0.01 | 0.03 |
|  |  | Twin | Home environment | Parental discipline | 3326 | 1758 | | | 1568 | 3.19 (1.59) | 3.07 (1.57) | 3.33 (1.60) | 22.20 | <0.01 | 0.01 |
|  |  |  |  | CHAOS | 3394 | 1791 | | | 1603 | 4.50 (2.34) | 4.34 (2.30) | 4.68 (2.37) | 17.83 | <0.01 | 0.01 |
|  |  |  | P factor | SDQ (Prosocial) | 3371 | 1780 | | | 1591 | 7.93 (1.85) | 8.33 (1.64) | 7.48 (1.96) | 188.33 | <0.01 | 0.05 |
|  |  |  |  | SDQ (Hyperactivity) | 3370 | 1779 | | | 1591 | 3.92 (2.26) | 3.62 (2.16) | 4.25 (2.32) | 65.97 | <0.01 | 0.02 |
|  |  |  |  | SDQ (Conduct) | 3369 | 1780 | | | 1589 | 2.21 (1.84) | 1.99 (1.72) | 2.46 (1.93) | 56.96 | <0.01 | 0.02 |
|  |  |  |  | SDQ (Peer Problems) | 3354 | 1780 | | | 1574 | 1.92 (1.75) | 1.79 (1.70) | 2.06 (1.80) | 19.81 | <0.01 | 0.01 |
|  |  |  |  | SDQ (Anxiety) | 3368 | 1778 | | | 1590 | 3.30 (2.37) | 3.46 (2.41) | 3.13 (2.32) | 15.50 | <0.01 | 0.00 |
|  |  |  |  | CAST (Social) | 3249 | 1726 | | | 1523 | 4.38 (2.31) | 4.06 (2.25) | 4.74 (2.32) | 71.65 | <0.01 | 0.02 |
|  |  |  |  | CAST (Non-Social) | 3260 | 1734 | | | 1526 | 3.92 (2.10) | 3.83 (2.06) | 4.02 (2.14) | 6.59 | 0.01 | 0.00 |
|  |  |  |  | CAST (Communication) | 3255 | 1732 | | | 1523 | 3.43 (2.55) | 3.21 (2.50) | 3.68 (2.59) | 28.81 | <0.01 | 0.01 |
| **12** | | Parent | P factor | SDQ (Prosocial) | 5865 | 3081 | | | 2784 | 8.54 (1.65) | 8.78 (1.53) | 8.26 (1.73) | 146.83 | <0.01 | 0.02 |
|  |  |  |  | SDQ (Hyperactivity) | 5850 | 3072 | | | 2778 | 2.86 (2.29) | 2.37 (2.03) | 3.41 (2.42) | 314.83 | <0.01 | 0.05 |
|  |  |  |  | SDQ (Conduct) | 5850 | 3072 | | | 2778 | 1.34 (1.49) | 1.24 (1.40) | 1.46 (1.57) | 32.96 | <0.01 | 0.01 |
|  |  |  |  | SDQ (Peer Problems) | 5849 | 3072 | | | 2777 | 1.12 (1.55) | 1.00 (1.43) | 1.25 (1.66) | 37.34 | <0.01 | 0.01 |
|  |  |  |  | SDQ (Anxiety) | 5850 | 3072 | | | 2778 | 1.85 (1.95) | 1.98 (2.01) | 1.70 (1.87) | 29.24 | <0.01 | 0.00 |
|  |  |  |  | MFQ | 5848 | 3072 | | | 2776 | 1.18 (2.31) | 1.22 (2.39) | 1.14 (2.21) | 2.06 | 0.15 | 0.00 |
|  |  |  |  | APSD (Callous-Unemotional) | 5864 | 3081 | | | 2783 | 3.09 (1.93) | 2.90 (1.86) | 3.29 (1.98) | 62.02 | <0.01 | 0.01 |
|  |  |  |  | APSD (Narcissism) | 5863 | 3080 | | | 2783 | 1.51 (1.78) | 1.32 (1.63) | 1.72 (1.92) | 74.78 | <0.01 | 0.01 |
|  |  |  |  | APSD (Impulsivity) | 5847 | 3073 | | | 2774 | 2.57 (1.90) | 2.25 (1.78) | 2.93 (1.97) | 195.06 | <0.01 | 0.03 |
|  |  |  |  | CAST (Social) | 6211 | 3224 | | | 2987 | 1.64 (1.55) | 1.32 (1.36) | 1.99 (1.66) | 301.93 | <0.01 | 0.05 |
|  |  |  |  | CAST (Non-Social) | 6203 | 3223 | | | 2980 | 1.43 (1.32) | 1.31 (1.25) | 1.56 (1.38) | 59.88 | <0.01 | 0.01 |
|  |  |  |  | CAST (Communication) | 6212 | 3225 | | | 2987 | 1.96 (1.92) | 1.84 (1.79) | 2.08 (2.04) | 24.92 | <0.01 | 0.00 |
|  |  |  |  | CBRS (Hyperactivity-Impulsivity) | 5855 | 3078 | | | 2777 | 4.34 (4.49) | 3.66 (3.83) | 5.10 (5.02) | 152.99 | <0.01 | 0.03 |
|  |  |  |  | CBRS (Inattention) | 5859 | 3081 | | | 2778 | 5.63 (5.21) | 4.57 (4.59) | 6.81 (5.59) | 281.58 | <0.01 | 0.05 |
|  |  | Twin | Home environment | Parental discipline | 5863 | 3078 | | | 2785 | 3.13 (1.48) | 3.04 (1.45) | 3.23 (1.50) | 22.73 | <0.01 | 0.00 |
|  |  |  |  | CHAOS | 5867 | 3079 | | | 2788 | 4.01 (2.06) | 3.87 (2.06) | 4.16 (2.04) | 28.90 | <0.01 | 0.00 |
|  |  |  | P factor | SDQ (Prosocial) | 5841 | 3059 | | | 2782 | 7.45 (1.92) | 7.90 (1.74) | 6.95 (1.98) | 381.79 | <0.01 | 0.06 |
|  |  |  |  | SDQ (Hyperactivity) | 5839 | 3059 | | | 2780 | 3.55 (2.31) | 3.14 (2.14) | 4.01 (2.41) | 209.47 | <0.01 | 0.03 |
|  |  |  |  | SDQ (Conduct) | 5839 | 3059 | | | 2780 | 1.92 (1.66) | 1.68 (1.52) | 2.18 (1.77) | 135.82 | <0.01 | 0.02 |
|  |  |  |  | SDQ (Peer Problems) | 5840 | 3059 | | | 2781 | 1.37 (1.60) | 1.28 (1.57) | 1.47 (1.62) | 21.96 | <0.01 | 0.00 |
|  |  |  |  | SDQ (Anxiety) | 5837 | 3058 | | | 2779 | 2.22 (2.07) | 2.41 (2.13) | 2.02 (1.99) | 51.66 | <0.01 | 0.01 |
|  |  |  |  | MFQ | 5857 | 3073 | | | 2784 | 2.35 (3.37) | 2.32 (3.47) | 2.38 (3.26) | 0.51 | 0.48 | 0.00 |
| **16** | | Parent | P factor | SDQ (Prosocial) | 5122 | 2818 | | | 2304 | 8.23 (1.95) | 8.51 (1.80) | 7.88 (2.07) | 137.04 | <0.01 | 0.03 |
|  |  |  |  | SDQ (Hyperactivity) | 5116 | 2814 | | | 2302 | 2.29 (1.99) | 1.98 (1.81) | 2.68 (2.13) | 161.72 | <0.01 | 0.03 |
|  |  |  |  | SDQ (Conduct) | 5126 | 2820 | | | 2306 | 1.23 (1.38) | 1.19 (1.35) | 1.28 (1.41) | 5.43 | 0.02 | 0.00 |
|  |  |  |  | ARBQ | 5127 | 2818 | | | 2309 | 3.67 (4.32) | 4.27 (4.63) | 2.94 (3.78) | 123.15 | <0.01 | 0.02 |
|  |  |  |  | MFQ | 5123 | 2817 | | | 2306 | 1.03 (2.37) | 1.22 (2.69) | 0.78 (1.89) | 44.45 | <0.01 | 0.01 |
|  |  |  |  | CBRS (Impulsivity) | 5119 | 2815 | | | 2304 | 2.63 (3.52) | 2.47 (3.26) | 2.83 (3.81) | 13.27 | <0.01 | 0.00 |
|  |  |  |  | CBRS (Inattention) | 5120 | 2815 | | | 2305 | 4.29 (4.99) | 3.40 (4.30) | 5.39 (5.52) | 210.85 | <0.01 | 0.04 |
|  |  |  |  | ICUT (Callous) | 5122 | 2816 | | | 2306 | 4.75 (3.68) | 4.32 (3.46) | 5.27 (3.86) | 86.94 | <0.01 | 0.02 |
|  |  |  |  | ICUT (Unemotional) | 5127 | 2818 | | | 2309 | 5.20 (2.94) | 4.69 (2.79) | 5.84 (2.99) | 202.93 | <0.01 | 0.04 |
|  |  |  |  | ICUT (Uncaring) | 5127 | 2818 | | | 2309 | 7.68 (4.83) | 6.81 (4.57) | 8.74 (4.94) | 212.24 | <0.01 | 0.04 |
|  |  |  |  | AQ (Social) | 5125 | 2817 | | | 2308 | 7.36 (4.76) | 6.93 (4.57) | 7.87 (4.94) | 49.74 | <0.01 | 0.01 |
|  |  |  |  | AQ (Attention Switching) | 5122 | 2815 | | | 2307 | 8.31 (4.28) | 7.84 (4.09) | 8.88 (4.43) | 76.55 | <0.01 | 0.01 |
|  |  |  |  | AQ (Imagination) | 5101 | 2810 | | | 2291 | 4.39 (3.46) | 3.85 (3.20) | 5.05 (3.64) | 157.02 | <0.01 | 0.03 |
|  |  |  |  | AQ (Attention to Detail) | 5085 | 2810 | | | 2275 | 4.89 (3.54) | 4.51 (3.42) | 5.36 (3.63) | 71.98 | <0.01 | 0.01 |
|  |  | Twin | Home environment | Parental discipline | 2745 | 1023 | | | 1452 | 3.09 (1.33) | 3.06 (1.31) | 3.11 (1.35) | 1.02 | 0.31 | 0.00 |
|  |  |  |  | CHAOS | 2782 | 1602 | | | 1180 | 4.13 (2.04) | 4.15 (2.06) | 4.12 (2.02) | 0.11 | 0.74 | 0.00 |
|  |  |  | P factor | SDQ (Prosocial) | 5088 | 2808 | | | 2280 | 7.13 (1.95) | 7.61 (1.80) | 6.53 (1.96) | 415.23 | <0.01 | 0.08 |
|  |  |  |  | SDQ (Hyperactivity) | 5089 | 2808 | | | 2281 | 3.59 (2.31) | 3.57 (2.33) | 3.62 (2.30) | 0.67 | 0.41 | 0.00 |
|  |  |  |  | SDQ (Conduct) | 5089 | 2808 | | | 2281 | 1.65 (1.46) | 1.56 (1.43) | 1.77 (1.50) | 25.24 | <0.01 | 0.00 |
|  |  |  |  | SDQ (Peer Problems) | 5091 | 2809 | | | 2282 | 1.57 (1.52) | 1.52 (1.50) | 1.63 (1.54) | 6.60 | 0.01 | 0.00 |
|  |  |  |  | SDQ (Anxiety) | 5090 | 2808 | | | 2282 | 2.76 (2.26) | 3.41 (2.33) | 1.96 (1.88) | 582.80 | <0.01 | 0.10 |
|  |  |  |  | MFQ | 5097 | 2815 | | | 2282 | 3.69 (4.54) | 4.50 (5.05) | 2.70 (3.56) | 205.38 | <0.01 | 0.04 |
|  |  |  |  | AQ (Social) | 5087 | 2810 | | | 2277 | 7.05 (4.21) | 7.11 (4.36) | 6.97 (4.01) | 1.41 | 0.24 | 0.00 |
|  |  |  |  | AQ (Attention to Detail) | 5085 | 2810 | | | 2275 | 4.89 (3.54) | 4.51 (3.42) | 5.36 (3.63) | 71.98 | <0.01 | 0.01 |
|  | Note: *n* = number of participants, SD = standard deviation, Group difference= F statistic | | | | | | | | | | | | | | |

## **Table S2.** *Factor loadings for the p factor model across ages and raters.*

| **Model** | **Measure** | **Estimate** | **z-value** | **P (>\|z\|)** |
| --- | --- | --- | --- | --- |
| Parent p at 9 | SDQ (Hyperactivity) | 0.644 | 39.505 | ≤ 0.01 |
|  | SDQ (Conduct) | 0.726 | 46.449 | ≤ 0.01 |
|  | SDQ (Peer problems) | 0.479 | 27.640 | ≤ 0.01 |
|  | SDQ (emotional problems) | 0.445 | 25.594 | ≤ 0.01 |
|  | APSD (callous-unemotional) | 0.411 | 23.388 | ≤ 0.01 |
|  | APSD (narcissism) | 0.709 | 44.923 | ≤ 0.01 |
|  | APSD (impulsivity) | 0.759 | 49.466 | ≤ 0.01 |
|  | RPAQ (proactive aggression) | 0.570 | 34.062 | ≤ 0.01 |
|  | RPAQ (reactive aggression) | 0.635 | 38.885 | ≤ 0.01 |
|  | CAST (social) | 0.348 | 19.442 | ≤ 0.01 |
|  | CAST (non-social) | 0.319 | 17.782 | ≤ 0.01 |
|  | CAST (communication) | 0.602 | 36.391 | ≤ 0.01 |
|  | SDQ Prosocial | -0.345 | -19.344 | ≤ 0.01 |
| Twin p at 9 | SDQ (Hyperactivity) | 0.525 | 28.126 | ≤ 0.01 |
|  | SDQ (Conduct) | 0.588 | 32.001 | ≤ 0.01 |
|  | SDQ (Peer problems) | 0.541 | 29.381 | ≤ 0.01 |
|  | SDQ (emotional problems) | 0.609 | 33.963 | ≤ 0.01 |
|  | CAST (social) | 0.266 | 13.410 | ≤ 0.01 |
|  | CAST (non-social) | 0.538 | 28.372 | ≤ 0.01 |
|  | CAST (communication) | 0.721 | 40.168 | ≤ 0.01 |
|  | SDQ Prosocial | -0.169 | -8.422 | ≤ 0.01 |
| Parent p at 12 | SDQ (Hyperactivity) | 0.777 | 67.169 | ≤ 0.01 |
|  | SDQ (Conduct) | 0.647 | 52.363 | ≤ 0.01 |
|  | SDQ (Peer Problems) | 0.469 | 35.611 | ≤ 0.01 |
|  | SDQ (Emotional Problems) | 0.426 | 32.039 | ≤ 0.01 |
|  | MFQ | 0.532 | 41.250 | ≤ 0.01 |
|  | APSD (Callous-Unemotional) | 0.418 | 31.467 | ≤ 0.01 |
|  | APSD (Narcissism) | 0.575 | 45.131 | ≤ 0.01 |
|  | APSD (Impulsivity) | 0.769 | 66.267 | ≤ 0.01 |
|  | CAST (Social) | 0.262 | 19.490 | ≤ 0.01 |
|  | CAST (Non-Social) | 0.350 | 26.433 | ≤ 0.01 |
|  | CAST (Communication) | 0.617 | 50.730 | ≤ 0.01 |
|  | Conners (Hyperactivity) | 0.708 | 58.992 | ≤ 0.01 |
|  | Conners (Inattention) | 0.734 | 61.814 | ≤ 0.01 |
|  | SDQ Prosocial | -0.372 | -27.642 | ≤ 0.01 |
| Twin p at 12 | SDQ (Hyperactivity) | 0.559 | 38.994 | ≤ 0.01 |
|  | SDQ (Conduct) | 0.601 | 42.149 | ≤ 0.01 |
|  | SDQ (Peer Problems) | 0.528 | 38.043 | ≤ 0.01 |
|  | SDQ (Emotional Problems) | 0.651 | 48.062 | ≤ 0.01 |
|  | MFQ | 0.753 | 56.459 | ≤ 0.01 |
|  | SDQ Prosocial | -0.213 | -14.054 | ≤ 0.01 |
| Parent p at 16 | SDQ (Hyperactivity) | 0.719 | 56.015 | ≤ 0.01 |
|  | SDQ (Conduct) | 0.640 | 48.371 | ≤ 0.01 |
|  | ARBQ Anxiety | 0.480 | 33.789 | ≤ 0.01 |
|  | MFQ | 0.502 | 35.897 | ≤ 0.01 |
|  | Conners hyperactivity | 0.485 | 34.360 | ≤ 0.01 |
|  | Conners inattention | 0.728 | 56.857 | ≤ 0.01 |
|  | ICUT (Callous) | 0.593 | 43.659 | ≤ 0.01 |
|  | ICUT (Unemotional) | 0.392 | 27.077 | ≤ 0.01 |
|  | ICUT (Uncaring) | 0.715 | 55.258 | ≤ 0.01 |
|  | AQ (Social) | 0.441 | 30.594 | ≤ 0.01 |
|  | AQ (Attention Switching) | 0.576 | 41.908 | ≤ 0.01 |
|  | AQ (Imagination) | 0.477 | 33.919 | ≤ 0.01 |
|  | AQ (Attention to Detail) | 0.011 | 0.700 | 0.484 |
|  | SDQ Prosocial | -0.595 | -43.596 | ≤ 0.01 |
| Twin p at 16 | SDQ (Hyperactivity) | 0.453 | 30.149 | ≤ 0.01 |
|  | SDQ (Conduct) | 0.402 | 26.315 | ≤ 0.01 |
|  | SDQ (Peer Problems) | 0.527 | 35.061 | ≤ 0.01 |
|  | SDQ (Emotional Problems) | 0.762 | 56.379 | ≤ 0.01 |
|  | MFQ | 0.773 | 56.923 | ≤ 0.01 |
|  | AQ (Social) | 0.500 | 32.637 | ≤ 0.01 |
|  | AQ (Attention to Detail) | 0.206 | 13.250 | ≤ 0.01 |
|  | SDQ Prosocial | -0.214 | -13.410 | ≤ 0.01 |

## **Table S3.** *Model fit statistics for the p factor model across ages and raters.*

| **Model fit** | **Parent p at 9** | **Twin p at 9** | **Parent p at 12** | **Twin p at 12** | **Parent p at 16** | **Twin p at 16** |
| --- | --- | --- | --- | --- | --- | --- |
| AIC | 115000.041 | 71502.478 | 213828.906 | 93213.877 | 186465.404 | 109163.31 |
| BIC | 115239.308 | 71649.564 | 214111.724 | 93334.065 | 186740.253 | 109320.207 |
| CFI | 0.75 | 0.756 | 0.797 | 0.823 | 0.687 | 0.172 |
| RMSA | 0.127 | 0.138 | 0.108 | 0.16 | 0.139 | 0.09 |
| SRMR | 0.08 | 0.078 | 0.068 | 0.068 | 0.087 | 0.067 |

|  |  |
| --- | --- |

## **Table S****4.** *Model fit statistics for phenotypic and genetic cross-lagged panel models*

| **model** | **ep** | **minus2LL** | **df** | **AIC** | **BIC** | **CFI** | **TLI** | **RMSEA** | **diffLL** | **diffdf** | **p** |
| --- | --- | --- | --- | --- | --- | --- | --- | --- | --- | --- | --- |
| Twin p to home environment (phenotypic) | 23 | 68944.1 | 25723 | 17498.1 | -166034.8 | 0.993 | 0.975 | 0.027 | 31.779 | 4 | <0.001 |
| Parent p to home environment (phenotypic) | 23 | 68001.8 | 26121 | 15759.8 | -170612.8 | 0.992 | 0.968 | 0.038 | 56.655 | 4 | <0.001 |
| Twin p to home environment (MZ differences) | 23 | 24373.2 | 9259 | 5855.2 | -50335.1 | 0.972 | 0.896 | 0.026 | 12.538 | 4 | 0.014 |
| Parent p to home environment (MZ differences) | 23 | 18452.8 | 9400 | -347.2 | -57393.2 | 0.995 | 0.983 | 0.012 | 5.900 | 4 | 0.207 |
| Twin p to home environment (genetic) | 57 | 132805.9 | 51714 | 29377.9 | -338785.0 | 0.997 | 0.996 | 0.006 | 169.138 | 123 | 0.004 |
| Parent p to home environment (genetic) | 57 | 124488.0 | 52482 | 19524.0 | -354106.4 | 0.997 | 0.996 | 0.008 | 203.753 | 123 | <0.001 |
| Twin p to CHAOS (phenotypic) | 23 | 69096.2 | 25695 | 17706.2 | -165626.8 | 0.983 | 0.937 | 0.042 | 70.201 | 4 | <0.001 |
| Parent p to CHAOS (phenotypic) | 23 | 68033.5 | 26093 | 15847.5 | -170325.3 | 0.986 | 0.946 | 0.049 | 91.864 | 4 | <0.001 |
| Twin p to CHAOS (MZ differences) | 23 | 24140.0 | 9245 | 5650.0 | -50455.2 | 0.985 | 0.945 | 0.018 | 8.146 | 4 | 0.086 |
| Parent p to CHAOS (MZ differences) | 23 | 18198.9 | 9386 | -573.1 | -57534.1 | 0.989 | 0.959 | 0.019 | 8.605 | 4 | 0.072 |
| Twin p to CHAOS (genetic) | 57 | 131068.1 | 51157 | 28754.1 | -335443.4 | 0.999 | 0.999 | 0.003 | 131.431 | 123 | 0.285 |
| Parent p to CHAOS (genetic) | 57 | 122515.5 | 51925 | 18665.5 | -350999.5 | 0.997 | 0.997 | 0.008 | 187.882 | 123 | <0.001 |
| Twin p to parental discipline (phenotypic) | 23 | 69723.8 | 25481 | 18761.8 | -163044.4 | 0.992 | 0.970 | 0.024 | 25.361 | 4 | <0.001 |
| Parent p to parental discipline (phenotypic) | 23 | 68298.2 | 25879 | 16540.2 | -168105.7 | 0.990 | 0.963 | 0.037 | 55.123 | 4 | <0.001 |
| Twin p to parental discipline (MZ differences) | 23 | 25256.3 | 9161 | 6934.3 | -48661.2 | 0.902 | 0.632 | 0.038 | 22.692 | 4 | <0.001 |
| Parent p to parental discipline (MZ differences) | 23 | 19206.4 | 9302 | 602.4 | -55848.8 | 1.003 | 1.012 | 0.000 | 2.677 | 4 | 0.613 |
| Twin p to parental discipline (genetic) | 57 | 135704.0 | 51228 | 33248.0 | -331455.0 | 0.995 | 0.994 | 0.007 | 176.938 | 123 | 27.001 |
| Parent p to parental discipline (genetic) | 57 | 126349.9 | 51996 | 22357.9 | -347812.6 | 0.998 | 0.998 | 0.007 | 172.529 | 123 | 0.002 |

Note: minus2LL = Minus 2*log-likelihood of the comparison model, df = degrees of freedom, AIC = Akaike information criterion, BIC = Bayesian information criterion.

## **Table S5.** *Cross-twin and cross-trait correlations.*

|  | **Twin correlations within trait** | |  | **Cross correlations** | |
| --- | --- | --- | --- | --- | --- |
| **Trait 1** | **MZ** | **DZ** | **Trait 2** | **MZ** | **DZ** |
| Twin p at 9 | 0.607 [0.570, 0.641] | 0.380 [0.343, 0.416] | Parent p at 9 | 0.426 [0.379, 0.470] | 0.305 [0.265, 0.343] |
|  |  |  | Home environment at 9 | 0.375 [0.326, 0.422] | 0.262 [0.222, 0.301] |
|  |  |  | CHAOS at 9 | 0.365 [0.316, 0.412] | 0.254 [0.213, 0.293] |
|  |  |  | Parental discipline at 9 | 0.267 [0.214, 0.319] | 0.181 [0.139, 0.222] |
|  |  |  | Twin p at 12 | 0.404 [0.351, 0.454] | 0.266 [0.221, 0.310] |
|  |  |  | Parent p at 12 | 0.392 [0.338, 0.442] | 0.272 [0.227, 0.316] |
|  |  |  | Home environment at 12 | 0.289 [0.231, 0.344] | 0.223 [0.177, 0.268] |
|  |  |  | CHAOS at 12 | 0.293 [0.235, 0.348] | 0.206 [0.160, 0.252] |
|  |  |  | Parental discipline at 12 | 0.179 [0.119, 0.238] | 0.161 [0.113, 0.207] |
|  |  |  | Twin p at 16 | 0.278 [0.212, 0.341] | 0.150 [0.096, 0.204] |
|  |  |  | Parent p at 16 | 0.291 [0.226, 0.353] | 0.219 [0.167, 0.271] |
|  |  |  | Home environment at 16 | 0.201 [0.127, 0.273] | 0.113 [0.053, 0.173] |
|  |  |  | CHAOS at 16 | 0.248 [0.176, 0.319] | 0.160 [0.100, 0.219] |
|  |  |  | Parental discipline at 16 | 0.060 [-0.019, 0.138] | 0.022 [-0.041, 0.085] |
| Parent p at 9 | 0.875 [0.861, 0.887] | 0.609 [0.582, 0.635] | Home environment at 9 | 0.338 [0.287, 0.386] | 0.282 [0.242, 0.321] |
|  |  |  | CHAOS at 9 | 0.355 [0.305, 0.402] | 0.284 [0.244, 0.323] |
|  |  |  | Parental discipline at 9 | 0.212 [0.158, 0.265] | 0.179 [0.137, 0.220] |
|  |  |  | Twin p at 12 | 0.325 [0.269, 0.379] | 0.290 [0.246, 0.334] |
|  |  |  | Parent p at 12 | 0.673 [0.638, 0.705] | 0.457 [0.418, 0.494] |
|  |  |  | Home environment at 12 | 0.328 [0.272, 0.381] | 0.281 [0.236, 0.324] |
|  |  |  | CHAOS at 12 | 0.320 [0.264, 0.374] | 0.249 [0.203, 0.293] |
|  |  |  | Parental discipline at 12 | 0.210 [0.150, 0.268] | 0.209 [0.163, 0.255] |
|  |  |  | Twin p at 16 | 0.197 [0.129, 0.263] | 0.174 [0.121, 0.227] |
|  |  |  | Parent p at 16 | 0.517 [0.464, 0.566] | 0.405 [0.359, 0.450] |
|  |  |  | Home environment at 16 | 0.185 [0.111, 0.258] | 0.194 [0.135, 0.252] |
|  |  |  | CHAOS at 16 | 0.233 [0.160, 0.304] | 0.239 [0.180, 0.295] |
|  |  |  | Parental discipline at 16 | 0.046 [-0.033, 0.124] | 0.070 [0.007, 0.132] |
| Home environment at 9 | 0.622 [0.587, 0.655] | 0.497 [0.464, 0.528] | CHAOS at 9 | 0.561 [0.521, 0.598] | 0.460 [0.425, 0.493] |
|  |  |  | Parental discipline at 9 | 0.473 [0.428, 0.516] | 0.354 [0.316, 0.391] |
|  |  |  | Twin p at 12 | 0.269 [0.210, 0.325] | 0.222 [0.175, 0.267] |
|  |  |  | Parent p at 12 | 0.322 [0.266, 0.376] | 0.204 [0.158, 0.250] |
|  |  |  | Home environment at 12 | 0.397 [0.344, 0.448] | 0.349 [0.306, 0.390] |
|  |  |  | CHAOS at 12 | 0.361 [0.306, 0.413] | 0.318 [0.274, 0.361] |
|  |  |  | Parental discipline at 12 | 0.287 [0.229, 0.343] | 0.251 [0.205, 0.295] |
|  |  |  | Twin p at 16 | 0.128 [0.059, 0.196] | 0.109 [0.055, 0.163] |
|  |  |  | Parent p at 16 | 0.269 [0.203, 0.332] | 0.184 [0.131, 0.236] |
|  |  |  | Home environment at 16 | 0.250 [0.177, 0.320] | 0.202 [0.143, 0.259] |
|  |  |  | CHAOS at 16 | 0.263 [0.191, 0.333] | 0.234 [0.176, 0.291] |
|  |  |  | Parental discipline at 16 | 0.135 [0.057, 0.212] | 0.089 [0.026, 0.151] |
| CHAOS at 9 | 0.638 [0.604, 0.670] | 0.513 [0.481, 0.544] | Parental discipline at 9 | 0.252 [0.199, 0.304] | 0.201 [0.159, 0.242] |
|  |  |  | Twin p at 12 | 0.274 [0.216, 0.331] | 0.220 [0.173, 0.265] |
|  |  |  | Parent p at 12 | 0.327 [0.271, 0.381] | 0.204 [0.157, 0.249] |
|  |  |  | Home environment at 12 | 0.382 [0.328, 0.433] | 0.336 [0.292, 0.377] |
|  |  |  | CHAOS at 12 | 0.424 [0.372, 0.473] | 0.363 [0.321, 0.404] |
|  |  |  | Parental discipline at 12 | 0.194 [0.134, 0.252] | 0.184 [0.137, 0.230] |
|  |  |  | Twin p at 16 | 0.136 [0.066, 0.204] | 0.077 [0.022, 0.132] |
|  |  |  | Parent p at 16 | 0.265 [0.199, 0.328] | 0.187 [0.133, 0.239] |
|  |  |  | Home environment at 16 | 0.209 [0.135, 0.281] | 0.195 [0.136, 0.253] |
|  |  |  | CHAOS at 16 | 0.284 [0.213, 0.353] | 0.277 [0.220, 0.333] |
|  |  |  | Parental discipline at 16 | 0.034 [-0.045, 0.112] | 0.033 [-0.031, 0.095] |
| Parental discipline at 9 | 0.521 [0.479, 0.561] | 0.385 [0.347, 0.421] | Twin p at 12 | 0.157 [0.096, 0.217] | 0.145 [0.097, 0.192] |
|  |  |  | Parent p at 12 | 0.198 [0.138, 0.256] | 0.125 [0.077, 0.172] |
|  |  |  | Home environment at 12 | 0.262 [0.204, 0.319] | 0.233 [0.186, 0.278] |
|  |  |  | CHAOS at 12 | 0.158 [0.097, 0.218] | 0.155 [0.107, 0.202] |
|  |  |  | Parental discipline at 12 | 0.273 [0.215, 0.330] | 0.223 [0.177, 0.269] |
|  |  |  | Twin p at 16 | 0.076 [0.006, 0.145] | 0.107 [0.052, 0.162] |
|  |  |  | Parent p at 16 | 0.174 [0.106, 0.241] | 0.114 [0.059, 0.168] |
|  |  |  | Home environment at 16 | 0.197 [0.122, 0.269] | 0.137 [0.076, 0.197] |
|  |  |  | CHAOS at 16 | 0.145 [0.069, 0.219] | 0.109 [0.048, 0.170] |
|  |  |  | Parental discipline at 16 | 0.184 [0.107, 0.260] | 0.112 [0.049, 0.175] |
| Twin p at 12 | 0.578 [0.548, 0.605] | 0.362 [0.333, 0.389] | Parent p at 12 | 0.404 [0.367, 0.439] | 0.277 [0.247, 0.306] |
|  |  |  | Home environment at 12 | 0.345 [0.307, 0.382] | 0.308 [0.278, 0.337] |
|  |  |  | CHAOS at 12 | 0.343 [0.305, 0.380] | 0.292 [0.263, 0.321] |
|  |  |  | Parental discipline at 12 | 0.216 [0.175, 0.257] | 0.209 [0.178, 0.239] |
|  |  |  | Twin p at 16 | 0.284 [0.236, 0.330] | 0.203 [0.165, 0.240] |
|  |  |  | Parent p at 16 | 0.322 [0.276, 0.367] | 0.232 [0.195, 0.269] |
|  |  |  | Home environment at 16 | 0.231 [0.159, 0.300] | 0.147 [0.089, 0.204] |
|  |  |  | CHAOS at 16 | 0.257 [0.186, 0.326] | 0.189 [0.132, 0.246] |
|  |  |  | Parental discipline at 16 | 0.112 [0.035, 0.187] | 0.032 [-0.029, 0.093] |
| Parent p at 12 | 0.855 [0.843, 0.865] | 0.519 [0.496, 0.541] | Home environment at 12 | 0.335 [0.296, 0.372] | 0.285 [0.255, 0.314] |
|  |  |  | CHAOS at 12 | 0.327 [0.288, 0.365] | 0.264 [0.234, 0.294] |
|  |  |  | Parental discipline at 12 | 0.215 [0.174, 0.256] | 0.198 [0.167, 0.229] |
|  |  |  | Twin p at 16 | 0.228 [0.179, 0.275] | 0.133 [0.095, 0.171] |
|  |  |  | Parent p at 16 | 0.600 [0.567, 0.632] | 0.376 [0.342, 0.409] |
|  |  |  | Home environment at 16 | 0.170 [0.097, 0.241] | 0.159 [0.101, 0.215] |
|  |  |  | CHAOS at 16 | 0.213 [0.141, 0.283] | 0.200 [0.142, 0.255] |
|  |  |  | Parental discipline at 16 | 0.049 [-0.028, 0.125] | 0.049 [-0.012, 0.109] |
| Home environment at 12 | 0.612 [0.584, 0.638] | 0.517 [0.493, 0.540] | CHAOS at 12 | 0.556 [0.525, 0.584] | 0.467 [0.441, 0.492] |
|  |  |  | Parental discipline at 12 | 0.438 [0.402, 0.471] | 0.371 [0.343, 0.398] |
|  |  |  | Twin p at 16 | 0.103 [0.052, 0.153] | 0.126 [0.087, 0.164] |
|  |  |  | Parent p at 16 | 0.224 [0.175, 0.272] | 0.195 [0.157, 0.232] |
|  |  |  | Home environment at 16 | 0.307 [0.238, 0.373] | 0.302 [0.248, 0.354] |
|  |  |  | CHAOS at 16 | 0.285 [0.215, 0.352] | 0.338 [0.284, 0.389] |
|  |  |  | Parental discipline at 16 | 0.221 [0.146, 0.293] | 0.142 [0.081, 0.201] |
| CHAOS at 12 | 0.631 [0.605, 0.656] | 0.554 [0.531, 0.575] | Parental discipline at 12 | 0.223 [0.182, 0.263] | 0.235 [0.204, 0.265] |
|  |  |  | Twin p at 16 | 0.154 [0.104, 0.203] | 0.129 [0.090, 0.167] |
|  |  |  | Parent p at 16 | 0.256 [0.208, 0.303] | 0.203 [0.165, 0.240] |
|  |  |  | Home environment at 16 | 0.255 [0.184, 0.324] | 0.277 [0.222, 0.330] |
|  |  |  | CHAOS at 16 | 0.343 [0.275, 0.407] | 0.364 [0.312, 0.414] |
|  |  |  | Parental discipline at 16 | 0.059 [-0.018, 0.135] | 0.071 [0.011, 0.132] |
| Parental discipline at 12 | 0.485 [0.452, 0.517] | 0.368 [0.340, 0.395] | Twin p at 16 | 0.014 [-0.037, 0.065] | 0.078 [0.039, 0.116] |
|  |  |  | Parent p at 16 | 0.109 [0.058, 0.159] | 0.115 [0.077, 0.154] |
|  |  |  | Home environment at 16 | 0.245 [0.174, 0.314] | 0.217 [0.161, 0.273] |
|  |  |  | CHAOS at 16 | 0.121 [0.046, 0.194] | 0.188 [0.131, 0.245] |
|  |  |  | Parental discipline at 16 | 0.300 [0.228, 0.368] | 0.161 [0.101, 0.219] |
| Twin p at 16 | 0.518 [0.484, 0.551] | 0.286 [0.254, 0.318] | Parent p at 16 | 0.311 [0.269, 0.352] | 0.212 [0.178, 0.244] |
|  |  |  | Home environment at 16 | 0.198 [0.130, 0.264] | 0.160 [0.106, 0.212] |
|  |  |  | CHAOS at 16 | 0.184 [0.116, 0.251] | 0.160 [0.107, 0.213] |
|  |  |  | Parental discipline at 16 | 0.148 [0.077, 0.218] | 0.091 [0.034, 0.147] |
| Parent p at 16 | 0.867 [0.856, 0.878] | 0.570 [0.546, 0.593] | Home environment at 16 | 0.251 [0.185, 0.315] | 0.217 [0.165, 0.269] |
|  |  |  | CHAOS at 16 | 0.264 [0.198, 0.328] | 0.232 [0.179, 0.283] |
|  |  |  | Parental discipline at 16 | 0.139 [0.068, 0.209] | 0.109 [0.052, 0.165] |
| Home environment at 16 | 0.537 [0.484, 0.585] | 0.429 [0.382, 0.474] | CHAOS at 16 | 0.517 [0.463, 0.567] | 0.477 [0.432, 0.519] |
|  |  |  | Parental discipline at 16 | 0.346 [0.280, 0.408] | 0.213 [0.157, 0.268] |
| CHAOS at 16 | 0.561 [0.511, 0.608] | 0.512 [0.468, 0.552] | Parental discipline at 16 | 0.244 [0.174, 0.311] | 0.178 [0.121, 0.233] |
| Parental discipline at 16 | 0.456 [0.395, 0.512] | 0.261 [0.205, 0.316] | - | - | - |

## **Table S6**. *Twin model-fitting results for univariate analyses parent-rated p factor and home environment. A = additive genetic, C = shared environmental, E = non-shared environmental proportions of the variance.*

##

| **Age** | **Composite** | **A** | **95% CI** | | **C** | **95% CI** | | **E** | **95% CI** | |
| --- | --- | --- | --- | --- | --- | --- | --- | --- | --- | --- |
|  |  |  | **Lower** | **Upper** |  | **Lower** | **Upper** |  | **Lower** | **Upper** |
| 9 | Parent p | 0.52 | 0.47 | 0.58 | 0.35 | 0.30 | 0.40 | 0.13 | 0.12 | 0.14 |
|  | Home environment | 0.19 | 0.13 | 0.27 | 0.41 | 0.35 | 0.46 | 0.39 | 0.36 | 0.42 |
| 12 | Parent p | 0.68 | 0.63 | 0.72 | 0.18 | 0.14 | 0.22 | 0.14 | 0.14 | 0.15 |
|  | Home environment | 0.19 | 0.12 | 0.24 | 0.42 | 0.38 | 0.47 | 0.39 | 0.36 | 0.41 |
| 16 | Parent p | 0.60 | 0.56 | 0.65 | 0.27 | 0.22 | 0.31 | 0.13 | 0.12 | 0.14 |
|  | Home environment | 0.31 | 0.21 | 0.41 | 0.26 | 0.18 | 0.33 | 0.44 | 0.40 | 0.48 |

## **Table S7.** *Percentages of genetic and environmental variance unique to each construct in parent-rated model after accounting for variance shared with previous time points. A = additive genetic, C = shared environmental, E = non-shared environmental proportions of the variance.*

| **Age** | **Composite** | **A(%)_spec** | **95% CI** | | **C(%)_spec** | **95% CI** | | **E(%)_spec** | **95% CI** | |
| --- | --- | --- | --- | --- | --- | --- | --- | --- | --- | --- |
|  |  |  | **Lower** | **Upper** |  | **Lower** | **Upper** |  | **Lower** | **Upper** |
| 9 | Parent p | 0.51 | 0.47 | 0.58 | 0.36 | 0.30 | 0.40 | 0.13 | 0.11 | 0.14 |
|  | Home environment | 0.19 | 0.13 | 0.27 | 0.41 | 0.35 | 0.46 | 0.39 | 0.36 | 0.42 |
| 12 | Parent p | 0.27 | 0.23 | 0.32 | 0.03 | 0.00 | 0.06 | 0.12 | 0.11 | 0.13 |
|  | Home environment | 0.12 | 0.02 | 0.20 | 0.19 | 0.12 | 0.24 | 0.39 | 0.36 | 0.41 |
| 16 | Parent p | 0.31 | 0.27 | 0.35 | 0.12 | 0.09 | 0.15 | 0.11 | 0.10 | 0.12 |
|  | Home environment | 0.26 | 0.17 | 0.35 | 0.09 | 0.02 | 0.18 | 0.43 | 0.39 | 0.46 |

## **Table S8.** *Twin model-fitting results for univariate analyses of twin-rated p factor and home environment. A = additive genetic, C = shared environmental, E = non-shared environmental proportions of the variance.*

| **Age** | **Composite** | **A** | **95% CI** | | **C** | **95% CI** | | **E** | **95% CI** | |
| --- | --- | --- | --- | --- | --- | --- | --- | --- | --- | --- |
|  |  |  | **Lower** | **Upper** |  | **Lower** | **Upper** |  | **Lower** | **Upper** |
| 9 | Twin p | 0.48 | 0.38 | 0.57 | 0.14 | 0.06 | 0.21 | 0.39 | 0.36 | 0.42 |
|  | Home environment | 0.20 | 0.13 | 0.28 | 0.41 | 0.35 | 0.46 | 0.39 | 0.36 | 0.42 |
| 12 | Twin p | 0.43 | 0.36 | 0.50 | 0.15 | 0.09 | 0.20 | 0.42 | 0.40 | 0.45 |
|  | Home environment | 0.20 | 0.15 | 0.26 | 0.42 | 0.37 | 0.45 | 0.39 | 0.36 | 0.40 |
| 16 | Twin p | 0.45 | 0.37 | 0.54 | 0.06 | 0.00 | 0.13 | 0.48 | 0.45 | 0.52 |
|  | Home environment | 0.29 | 0.19 | 0.39 | 0.27 | 0.19 | 0.35 | 0.44 | 0.40 | 0.48 |

## **Table S9.** *Percentages of genetic and environmental variance unique to each construct in twin-rated model after accounting for variance shared with previous time points. A = additive genetic, C = shared environmental, E = non-shared environmental proportions of the variance.*

| **Age** | **Composite** | **A(%)_spec** | **95% CI** | | **C(%)_spec** | **95% CI** | | **E(%)_spec** | **95% CI** | |
| --- | --- | --- | --- | --- | --- | --- | --- | --- | --- | --- |
|  |  |  | **Lower** | **Upper** |  | **Lower** | **Upper** |  | **Lower** | **Upper** |
| 9 | Twin p | 0.47 | 0.41 | 0.51 | 0.15 | 0.11 | 0.19 | 0.39 | 0.36 | 0.41 |
|  | Home environment | 0.20 | 0.13 | 0.28 | 0.41 | 0.35 | 0.46 | 0.39 | 0.36 | 0.42 |
| 12 | Twin p | 0.21 | 0.15 | 0.27 | 0.07 | 0.03 | 0.11 | 0.42 | 0.39 | 0.44 |
|  | Home environment | 0.15 | 0.09 | 0.22 | 0.12 | 0.04 | 0.18 | 0.38 | 0.36 | 0.40 |
| 16 | Twin p | 0.30 | 0.25 | 0.35 | 0.03 | 0.01 | 0.07 | 0.47 | 0.44 | 0.49 |
|  | Home environment | 0.24 | 0.15 | 0.33 | 0.10 | 0.03 | 0.17 | 0.43 | 0.39 | 0.47 |

## **Table S10.** *Twin model-fitting results for univariate analyses of parent- and twin-rated p factor, twin-rated CHAOS and twin-rated parental discipline. A = additive genetic, C = shared environmental, E = non-shared environmental proportions of the variance.*

##

| **Age** | **Composite** | **A** | **95% CI** | | **C** | **95% CI** | | **E** | **95% CI** | |
| --- | --- | --- | --- | --- | --- | --- | --- | --- | --- | --- |
|  |  |  | **Lower** | **Upper** |  | **Lower** | **Upper** |  | **Lower** | **Upper** |
| 9 | CHAOS | 0.23 | 0.15 | 0.31 | 0.40 | 0.33 | 0.46 | 0.37 | 0.34 | 0.40 |
|  | Parental discipline | 0.22 | 0.12 | 0.32 | 0.28 | 0.20 | 0.36 | 0.50 | 0.46 | 0.53 |
| 12 | CHAOS | 0.15 | 0.09 | 0.21 | 0.48 | 0.43 | 0.52 | 0.37 | 0.35 | 0.39 |
|  | Parental discipline | 0.24 | 0.16 | 0.32 | 0.25 | 0.19 | 0.31 | 0.51 | 0.48 | 0.54 |
| 16 | CHAOS | 0.14 | 0.04 | 0.25 | 0.43 | 0.34 | 0.52 | 0.42 | 0.38 | 0.47 |
|  | Parental discipline | 0.42 | 0.26 | 0.51 | 0.05 | 0.00 | 0.16 | 0.54 | 0.49 | 0.60 |

## **Table S11.** *Descriptive statistics for the MZ difference scores*

| **Age wave** | **Informant** | **Variable** | **Mean (SD)** | **Min** | **Max** | **Median** | **Skewness** | **Kurtosis** |
| --- | --- | --- | --- | --- | --- | --- | --- | --- |
| **9** | Parent | p factor | 0.02 (0.50) | -2.73 | 3.16 | 0.01 | 0.13 | 5.14 |
|  | Twin | p factor | 0.00 (0.88) | -3.84 | 4.43 | 0.00 | 0.04 | 0.97 |
|  |  | Home environment | 0.00 (0.73) | -2.91 | 2.50 | 0.04 | -0.14 | 1.00 |
|  |  | CHAOS | -0.01 (0.86) | -3.86 | 3.00 | 0.02 | -0.17 | 0.90 |
|  |  | Parental discipline | 0.02 (1.01) | -3.74 | 4.50 | 0.06 | 0.05 | 1.16 |
| **12** | Parent | p factor | 0.01 (0.54) | -2.64 | 2.69 | 0.00 | 0.26 | 3.55 |
|  | Twin | p factor | -0.01 (0.92) | -3.24 | 3.23 | 0.00 | -0.02 | 0.55 |
|  |  | Home environment | 0.00 (0.71) | -2.75 | 2.93 | 0.00 | -0.11 | 0.69 |
|  |  | CHAOS | -0.01 (0.86) | -3.94 | 3.41 | 0.00 | -0.09 | 1.17 |
|  |  | Parental discipline | 0.00 (1.01) | -4.00 | 4.03 | -0.01 | -0.07 | 0.53 |
| **16** | Parent | p factor | -0.02 (0.51) | -3.57 | 2.63 | 0.00 | -0.32 | 5.11 |
|  | Twin | p factor | -0.02 (0.99) | -4.14 | 3.25 | -0.02 | 0.02 | 0.55 |
|  |  | Home environment | -0.35 (0.71) | -2.55 | 2.91 | -0.35 | 0.32 | 0.84 |
|  |  | CHAOS | 0.02 (0.91) | -3.02 | 3.45 | -0.04 | 0.18 | 0.46 |
|  |  | Parental discipline | -0.75 (0.88) | -3.07 | 2.60 | -0.80 | 0.38 | 0.68 |

## **Table S12.** *A correlation matrix for MZ difference scores*.

|  | **Twin p at 9** | **Twin p at 12** | **Twin p at 16** | **Home environment at 9** | **Home environment at 12** | **Home environment at 16** | **CHAOS at 9** | **CHAOS at 12** | **CHAOS at 16** | **Parental discipline at 9** | **Parental discipline at 12** | **Parental discipline at 16** | **Parent p at 9** | **Parent p at 12** | **Parent p at 16** |
| --- | --- | --- | --- | --- | --- | --- | --- | --- | --- | --- | --- | --- | --- | --- | --- |
| **Twin p at 9** | 1.00 | 0.15 | 0.08 | 0.18 | 0.10 | 0.09 | 0.18 | 0.07 | 0.10 | 0.11 | 0.07 | 0.05 | 0.21 | 0.15 | 0.05 |
| **Twin p at 12** | 0.15 | 1.00 | 0.19 | 0.05 | 0.27 | 0.06 | 0.03 | 0.26 | 0.07 | 0.06 | 0.16 | 0.03 | 0.12 | 0.25 | 0.12 |
| **Twin p at 16** | 0.08 | 0.19 | 1.00 | 0.09 | 0.07 | 0.15 | -0.01 | 0.05 | 0.17 | 0.14 | 0.05 | 0.06 | 0.02 | 0.12 | 0.23 |
| **Home environment at 9** | 0.18 | 0.05 | 0.09 | 1.00 | 0.04 | 0.06 | 0.73 | -0.02 | 0.08 | 0.81 | 0.07 | 0.03 | 0.05 | 0.04 | 0.01 |
| **Home environment at 12** | 0.10 | 0.27 | 0.07 | 0.04 | 1.00 | 0.09 | 0.04 | 0.71 | 0.07 | 0.01 | 0.80 | 0.06 | -0.04 | 0.05 | 0.06 |
| **Home environment at 16** | 0.09 | 0.06 | 0.15 | 0.06 | 0.09 | 1.00 | 0.06 | 0.06 | 0.77 | 0.02 | 0.08 | 0.75 | 0.00 | 0.05 | 0.08 |
| **CHAOS at 9** | 0.18 | 0.03 | -0.01 | 0.73 | 0.04 | 0.06 | 1.00 | 0.01 | 0.07 | 0.17 | 0.04 | 0.06 | 0.03 | 0.03 | -0.01 |
| **CHAOS at 12** | 0.07 | 0.26 | 0.05 | -0.02 | 0.71 | 0.06 | 0.01 | 1.00 | 0.05 | -0.05 | 0.15 | 0.03 | -0.08 | 0.06 | 0.03 |
| **CHAOS at 16** | 0.10 | 0.07 | 0.17 | 0.08 | 0.07 | 0.77 | 0.07 | 0.05 | 1.00 | 0.04 | 0.05 | 0.14 | -0.04 | 0.05 | 0.07 |
| **Parental discipline at 9** | 0.11 | 0.06 | 0.14 | 0.81 | 0.01 | 0.02 | 0.17 | -0.05 | 0.04 | 1.00 | 0.05 | -0.01 | 0.03 | 0.03 | 0.01 |
| **Parental discipline at 12** | 0.07 | 0.16 | 0.05 | 0.07 | 0.80 | 0.08 | 0.04 | 0.15 | 0.05 | 0.05 | 1.00 | 0.06 | 0.02 | 0.02 | 0.05 |
| **Parental discipline at 16** | 0.05 | 0.03 | 0.06 | 0.03 | 0.06 | 0.75 | 0.06 | 0.03 | 0.14 | -0.01 | 0.06 | 1.00 | 0.05 | 0.04 | 0.05 |
| **Parent p at 9** | 0.21 | 0.12 | 0.02 | 0.05 | -0.04 | 0.00 | 0.03 | -0.08 | -0.04 | 0.03 | 0.02 | 0.05 | 1.00 | 0.44 | 0.17 |
| **Parent p at 12** | 0.15 | 0.25 | 0.12 | 0.04 | 0.05 | 0.05 | 0.03 | 0.06 | 0.05 | 0.03 | 0.02 | 0.04 | 0.44 | 1.00 | 0.33 |
| **Parent p at 16** | 0.05 | 0.12 | 0.23 | 0.01 | 0.06 | 0.08 | -0.01 | 0.03 | 0.07 | 0.01 | 0.05 | 0.05 | 0.17 | 0.33 | 1.00 |

# **Supplementary Figures**

## **Figure S1.** *A correlation heatmap for all phenotypic variables at age 9*


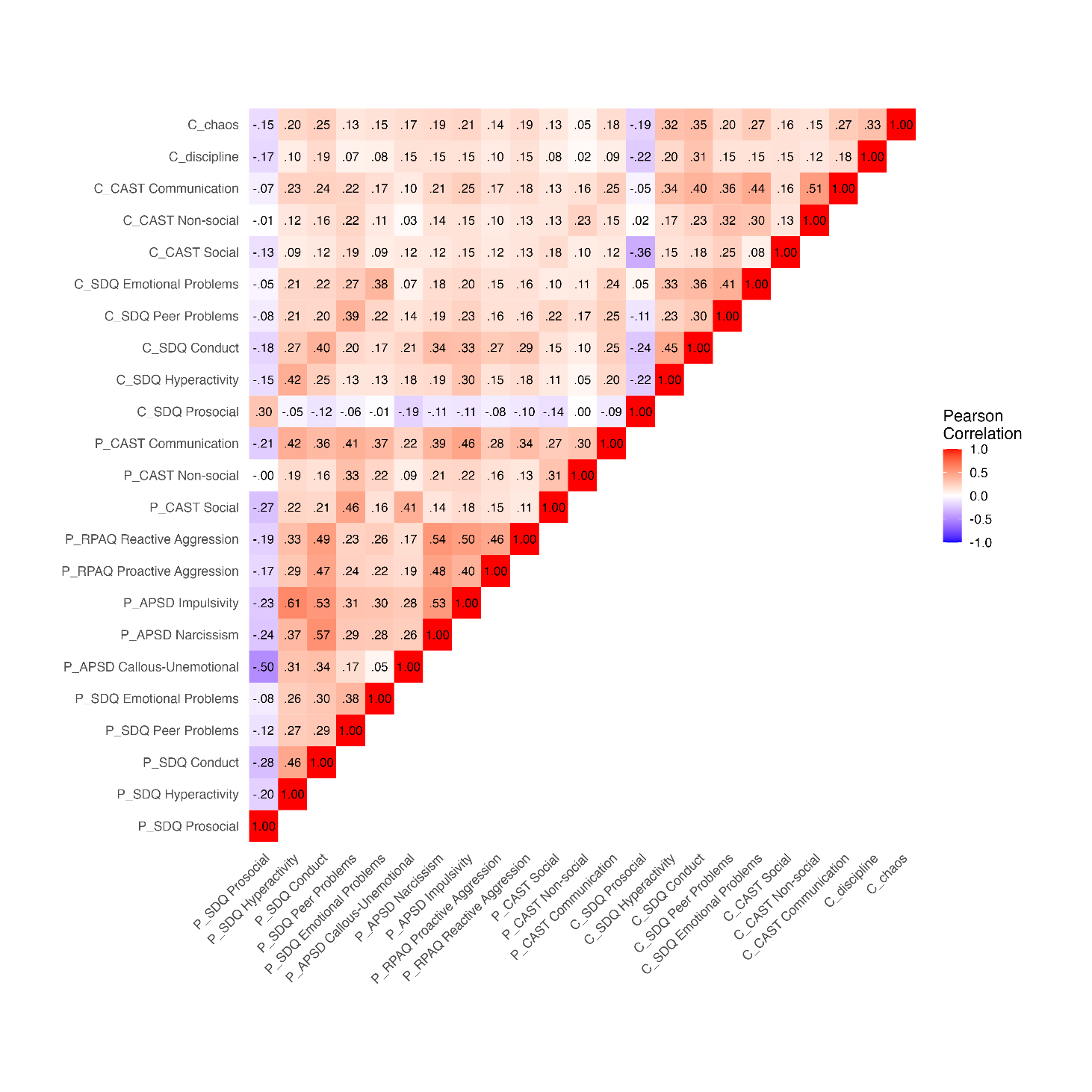


Note: Variable name preceded by P = parent report, C = child-report. CAST = Childhood Autism Spectrum Test, SDQ = Strengths and Difficulties Questionnaire, RPAQ = Reactive-Proactive Aggression Questionnaire, APSD = Anti-Social Process Screening Device.

## **Figure S2.** *A correlation heatmap for all phenotypic variables at age 12*


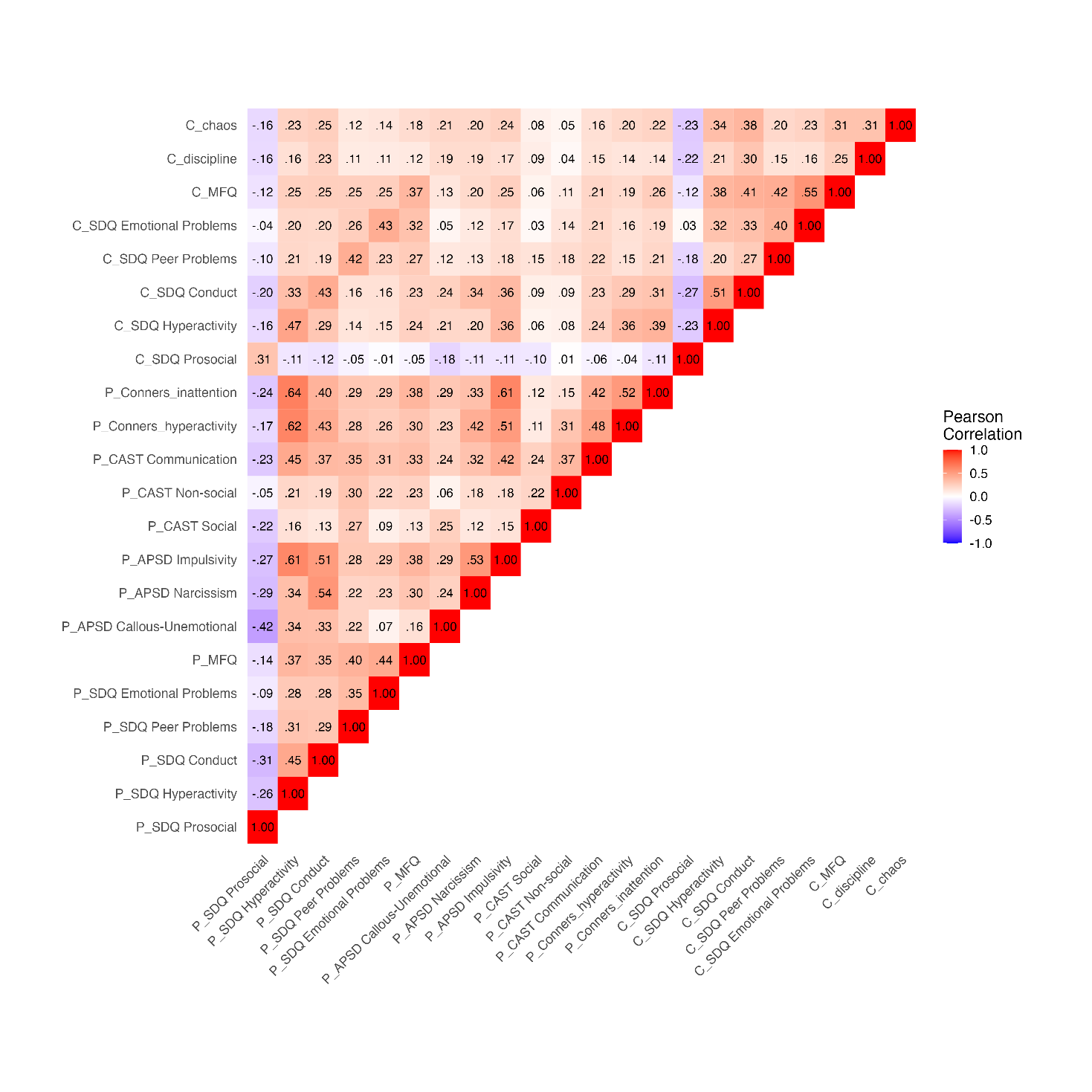


Note: Variable name preceded by P = parent report, C = child-report. SDQ = Strengths and Difficulties Questionnaire, CAST = Childhood Autism Spectrum Test, APSD = Anti-Social Process Screening Device, MFQ = Mood and Feelings Questionnaire.

## **Figure S3.** *A correlation heatmap for all phenotypic variables at age 16*


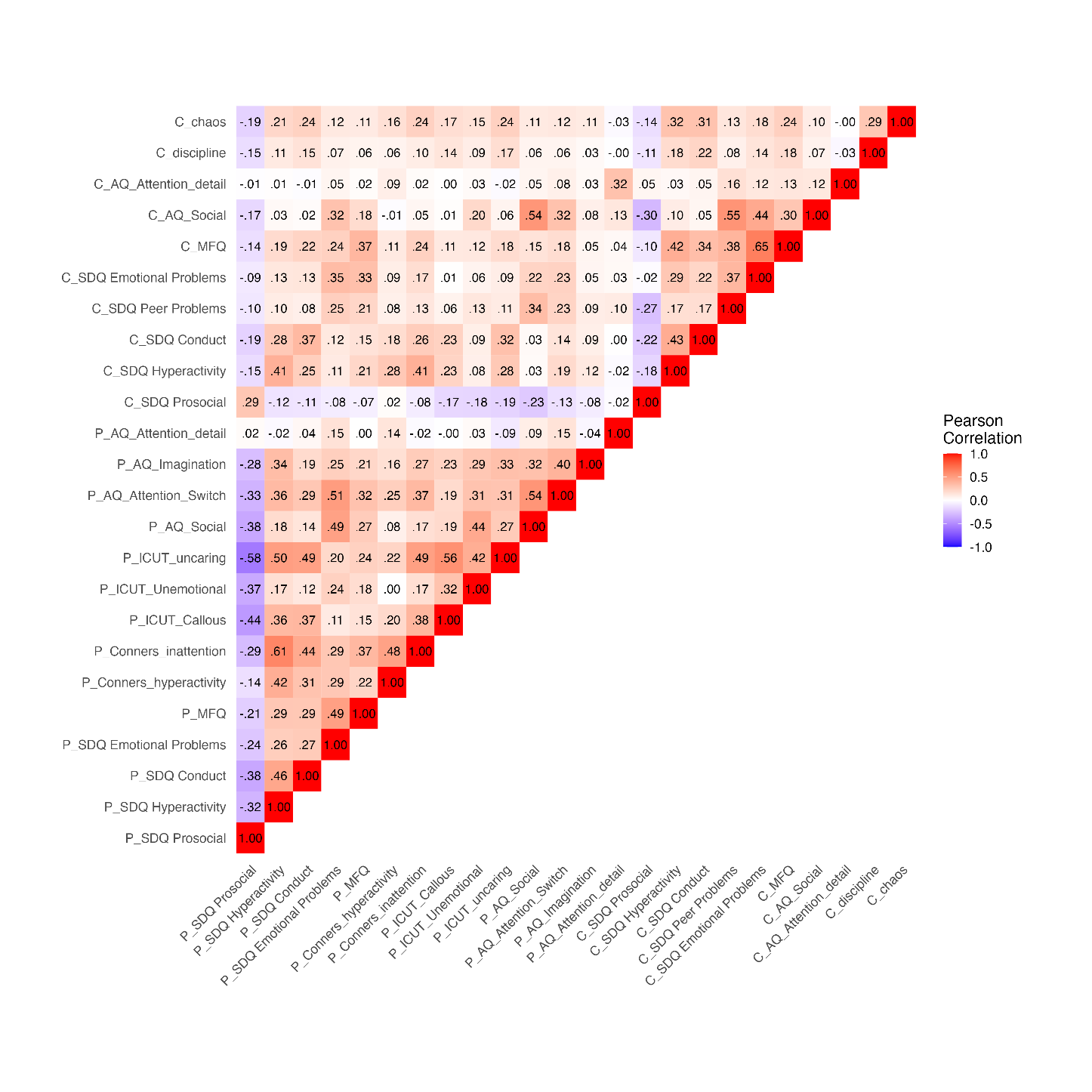


Note: Variable name preceded by P = parent report, C = child-report. AQ = Autism Quotient, MFQ = Mood and Feelings Questionnaire, SDQ = Strengths and Difficulties Questionnaire, ICUT = Inventory of Callous-Unemotional Traits.

## **Figure S4.** *A correlation heatmap for all composites*


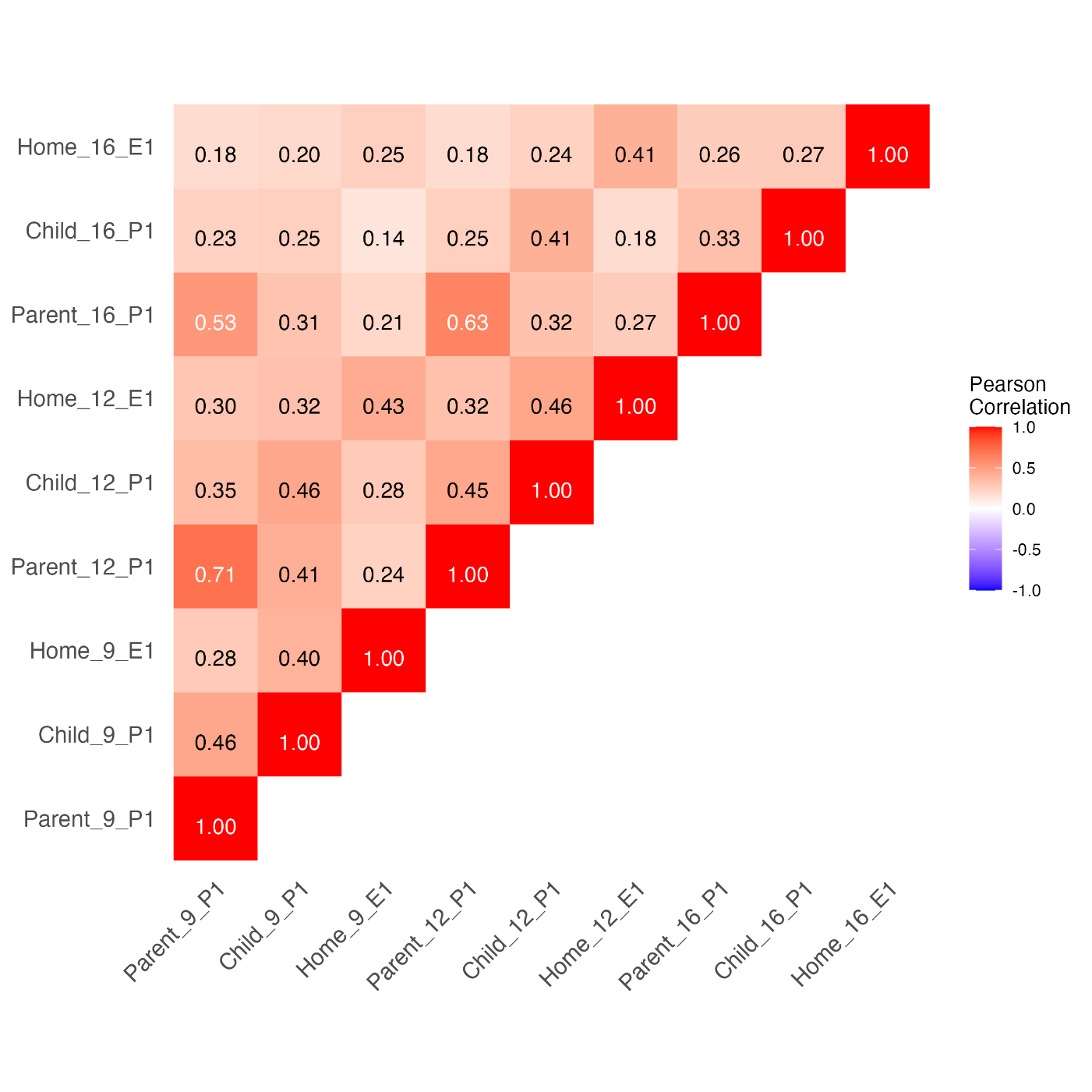


Note: ‘Parent’ and ‘Child’ indicate informant who completed constituent measures. Numbers 9, 12 and 16 denote measurement age. P1 = p factor (1^st^ principal component), E1 = home environment (1^st^ principal component).

## **Figure S5.** *A phenotypic cross-lagged panel model between the parent-rated p factor and twin-rated CHAOS.*

0.71

(0.69-0.73)

0.14

(0.11-0.17)

0.08

(0.05-0.10)

0.39

(0.36-0.42)

0.64

(0.62-0.66)

0.07

(0.05-0.09)

0.13

(0.08-0.17)

0.39

(0.35-0.43)

1.00

(1.00-1.00)

1.00

(1.00-1.00)

0.29

(0.26-0.32)

0.45

(0.43-0.48)

0.55

(0.53-0.58)

0.79

(0.77-0.82)

0.80

(0.77-0.83)

0.09

(0.07-0.11)

0.12

(0.09-0.15)

## **Figure S6.** Genetic and environmental decomposition of associations between parent-rated p factor and twin-rated CHAOS at ages 9, 12 and 16. ‘A’ represents the proportion of variance (%) explained by additive genetic effects, ‘C’ by shared environment and ‘E’ by non-shared environment*.*

**
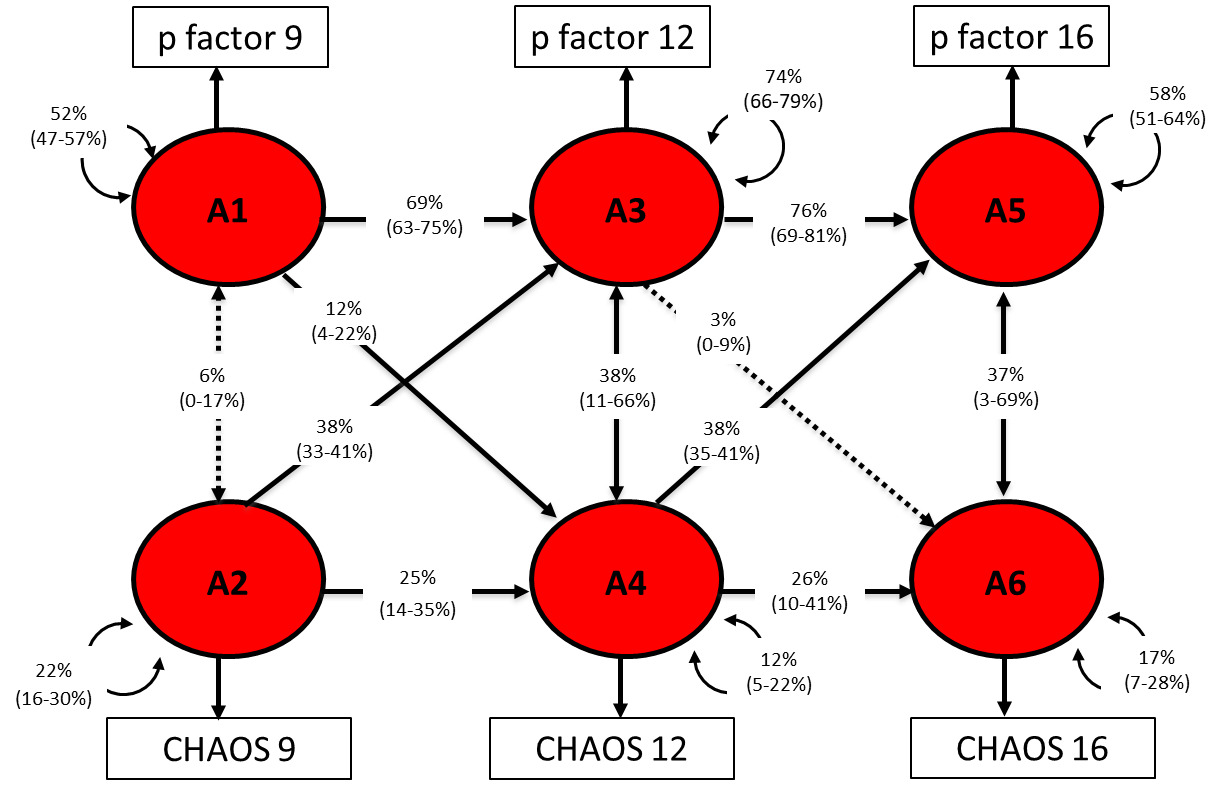
**

**
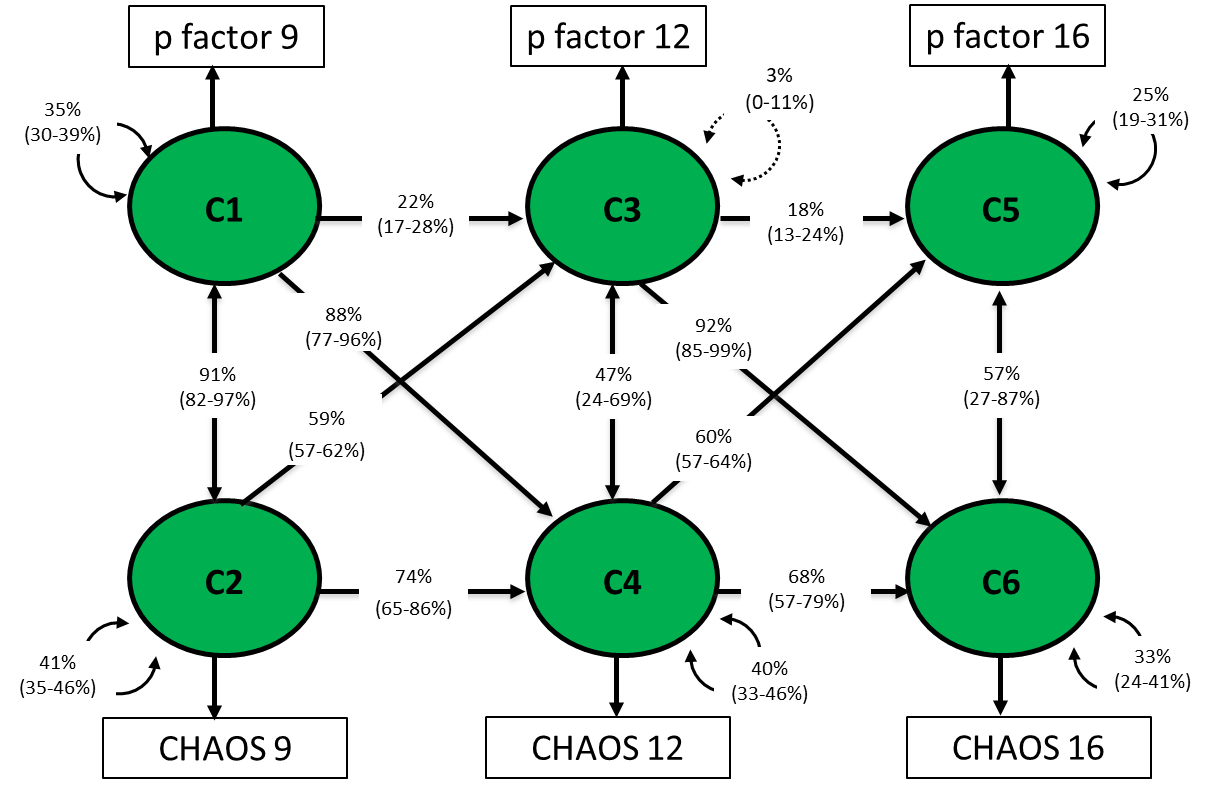
**

**
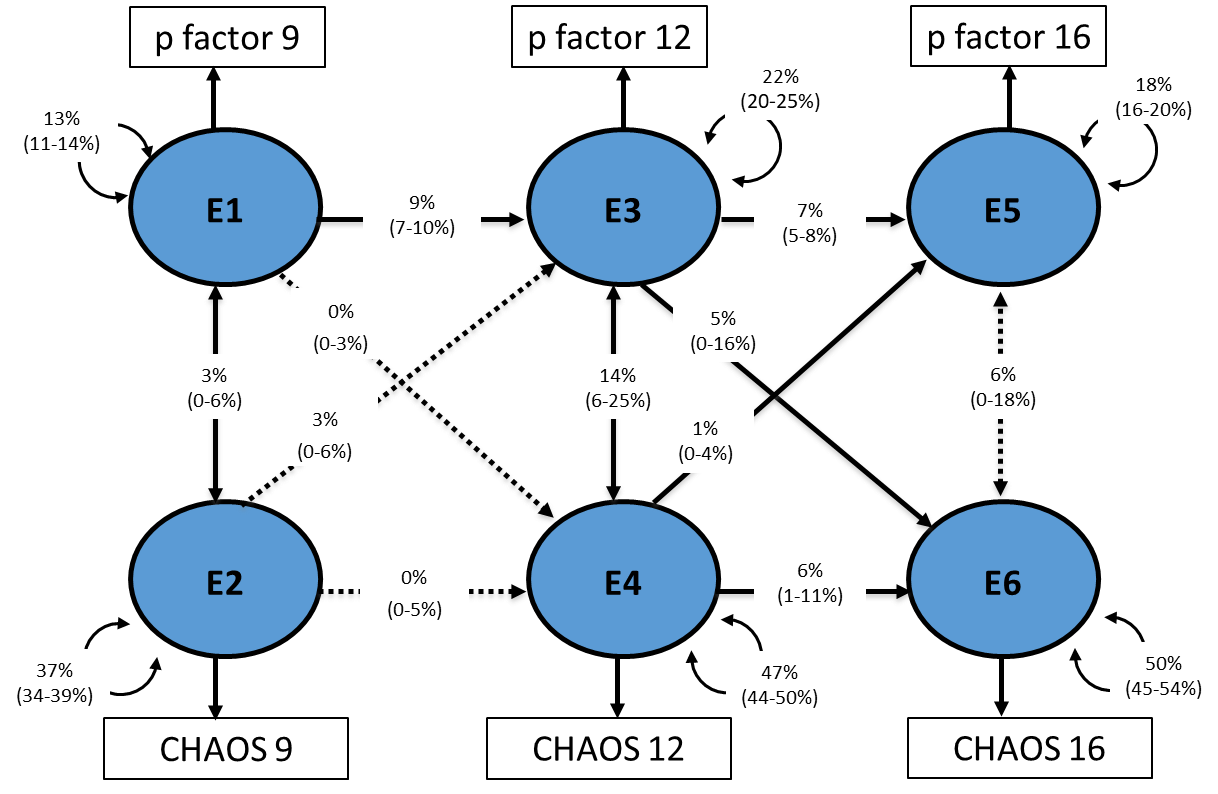
**

## **Figure S7.** *A phenotypic cross-lagged panel model between the twin-rated p factor and twin-rated CHAOS.*

0.43

(0.40-0.46)

0.12

(0.09-0.16)

0.11

(0.08-0.15)

0.39

(0.35-0.42)

0.39

(0.37-0.42)

0.02

(-0.01-0.05)

0.12

(0.08-0.17)

0.38

(0.34-0.42)

1.00

(1.00-1.00)

1.00

(1.00-1.00)

0.39

(0.36-0.41)

0.76

(0.74-0.79)

0.84

(0.82-0.86)

0.80

(0.77-0.83)

0.80

(0.77-0.83)

0.25

(0.23-0.27)

0.15

(0.12-0.19)

## **Figure S8.** Genetic and environmental decomposition of associations between twin-rated p factor and twin-rated CHAOS at ages 9, 12 and 16. ‘A’ represents the proportion of variance (%) explained by additive genetic effects, ‘C’ by shared environment and ‘E’ by non-shared environment*.*


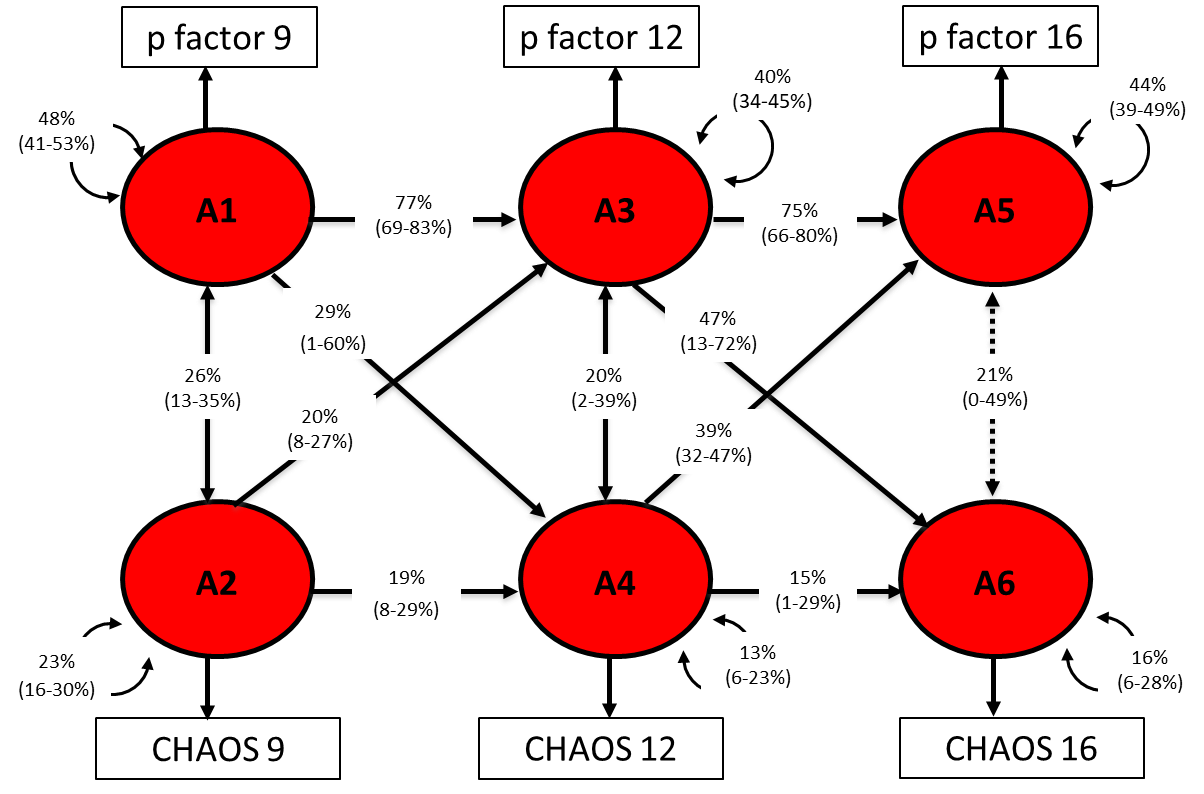


**
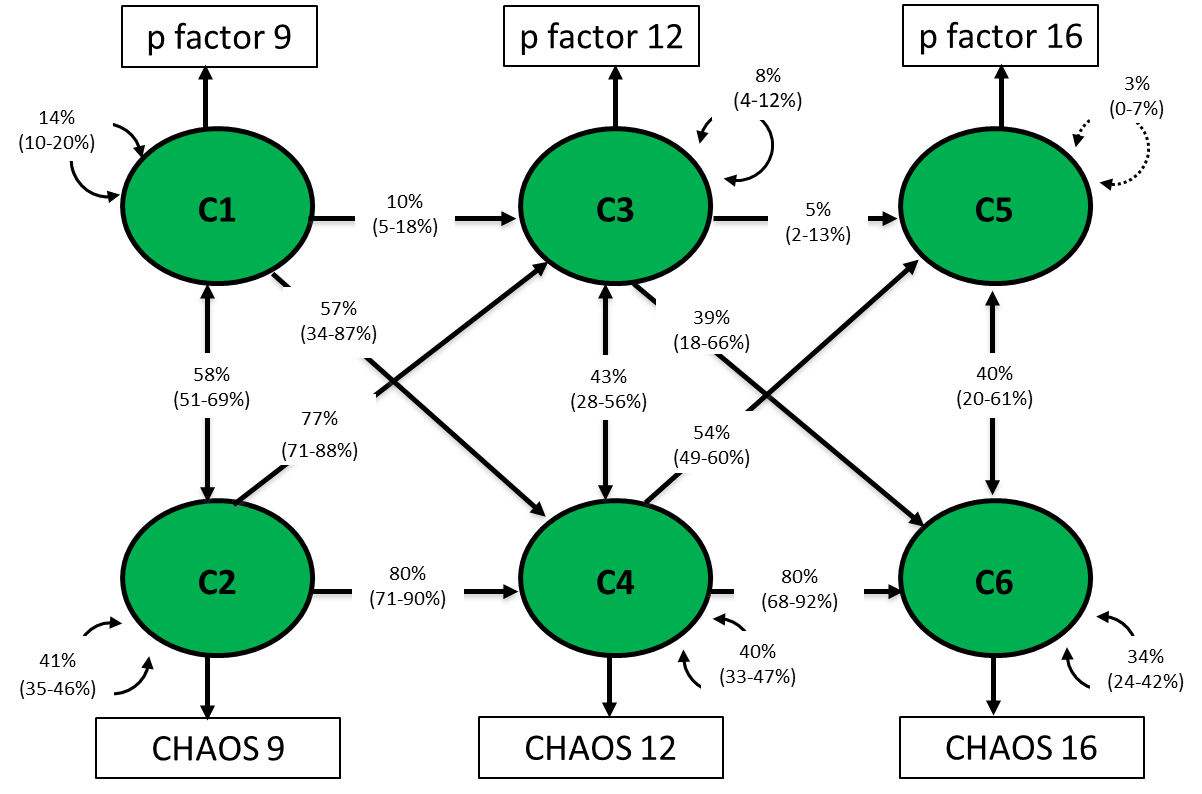
**

## **
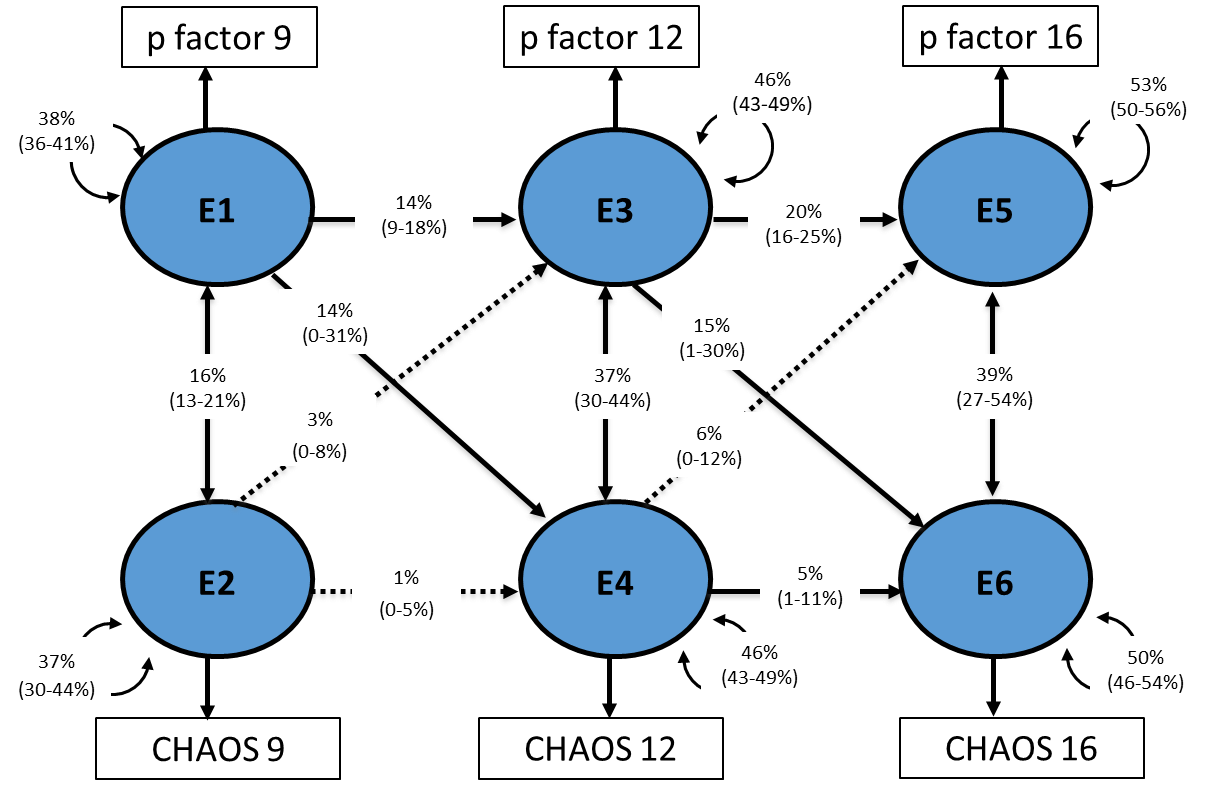
**

## **Figure S9.** *A phenotypic cross-lagged panel model between the parent-rated p factor and twin-rated parental discipline.*

0.74

(0.72-0.75)

0.17

(0.14-0.20)

-0.01

(-0.04-0.01)

0.30

(0.26-0.33)

0.65

(0.63-0.67)

0.06

(0.03-0.08)

0.04

(-0.01-0.08)

0.31

(0.26-0.35)

1.00

(1.00-1.00)

1.00

(1.00-1.00)

0.19

(0.16-0.23)

0.46

(0.44-0.48)

0.56

(0.53-0.58)

0.86

(0.84-0.89)

0.90

(0.87-0.92)

0.07

(0.04-0.09)

0.09

(0.06-0.12)

## **Figure S10.** *Genetic and environmental decomposition of associations between parent-rated p factor and twin-rated ‘Parental Discipline’ at ages 9, 12 and 16. ‘A’ represents the proportion of variance (%) explained by additive genetic effects, ‘C’ by shared environment and ‘E’ by non-shared environment.*


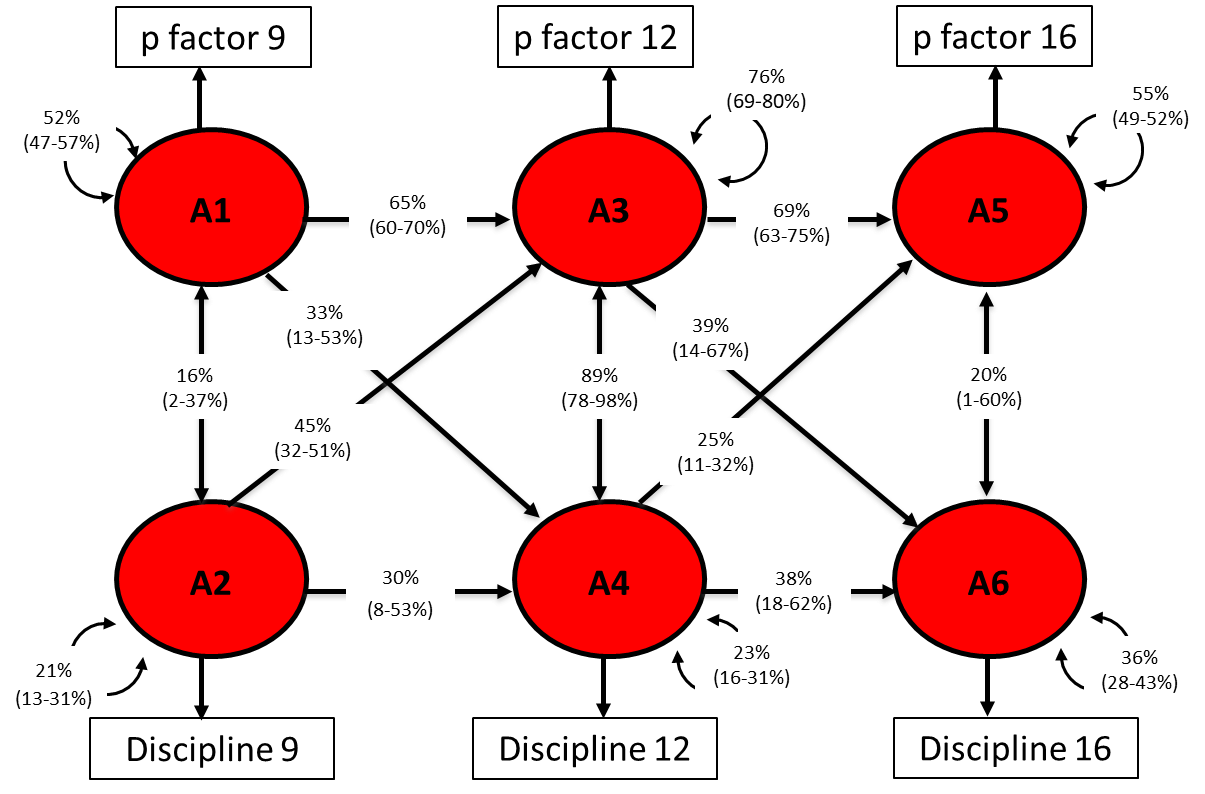


**
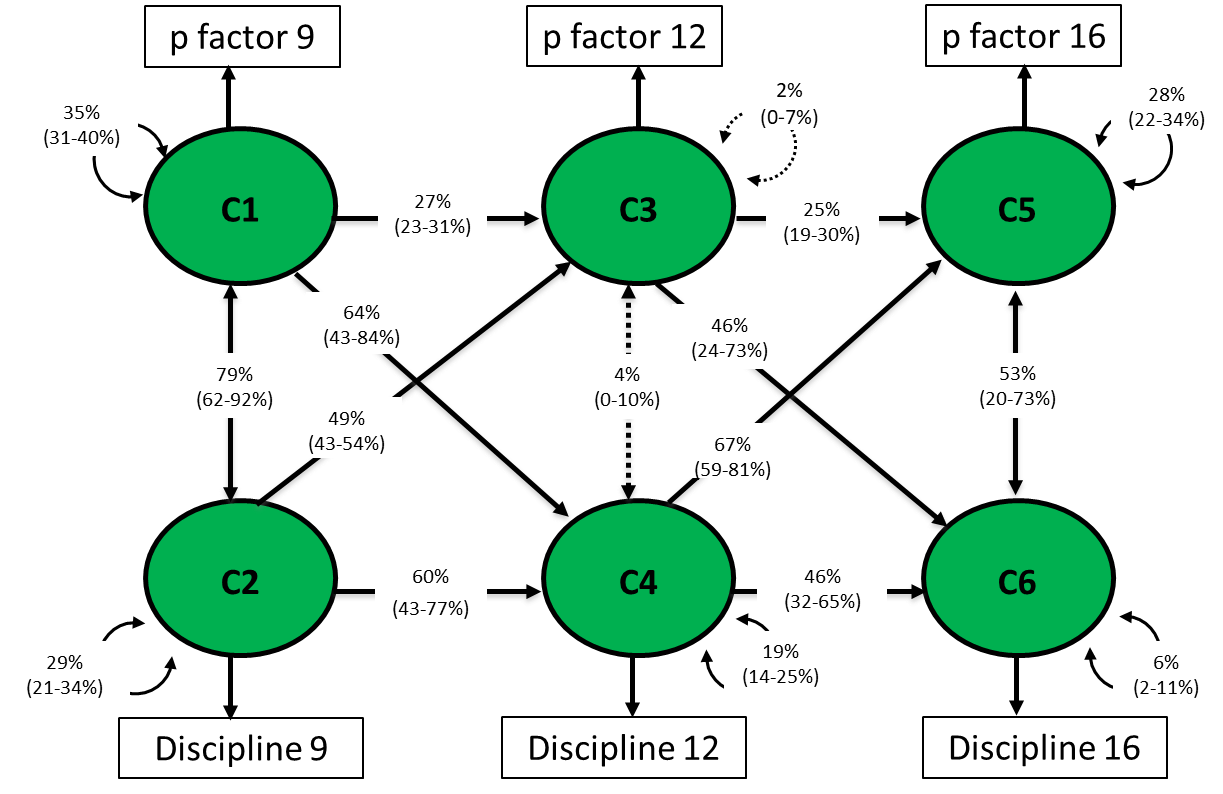
**

**
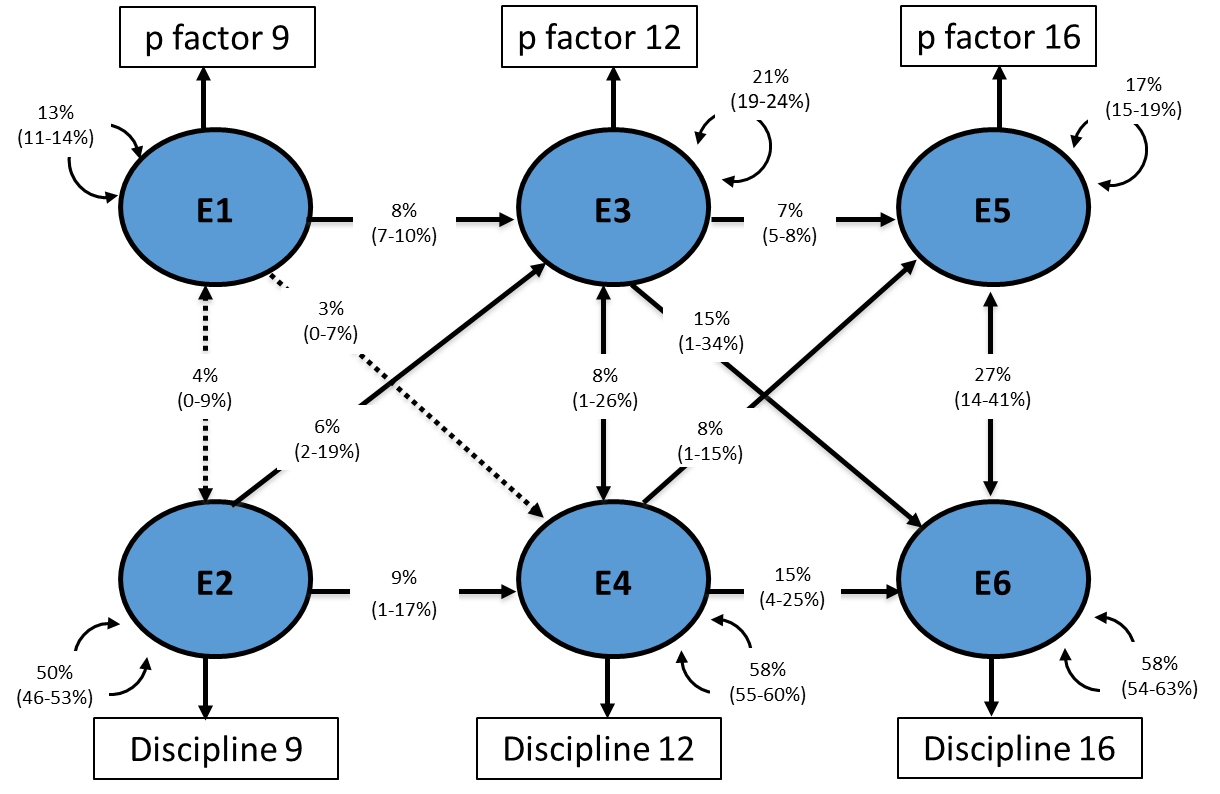
**

## **Figure S11.** *A phenotypic cross-lagged panel model between the twin-rated p factor and twin-rated parental discipline.*

0.46

(0.43-0.49)

0.13

(0.10-0.17)

0.05

(0.02-0.09)

0.29

(0.25-0.32)

0.40

(0.37-0.43)

0.00

(-0.03-0.03)

0.04

(-0.01-0.09)

0.30

(0.25-0.31)

1.00

(1.00-1.00)

1.00

(1.00-1.00)

0.28

(0.25-0.31)

0.77

(0.75-0.80)

0.84

(0.82-0.86)

0.88

(0.86-0.90)

0.90

(0.87-0.93)

0.19

(0.17-0.22)

0.14

(0.10-0.18)

## **Figure S12.** *Genetic and environmental decomposition of associations between twin-rated p factor and twin-rated ‘Parental Discipline’ at ages 9, 12 and 16. ‘A’ represents the proportion of variance (%) explained by additive genetic effects, ‘C’ by shared environment and ‘E’ by non-shared environment.*

**
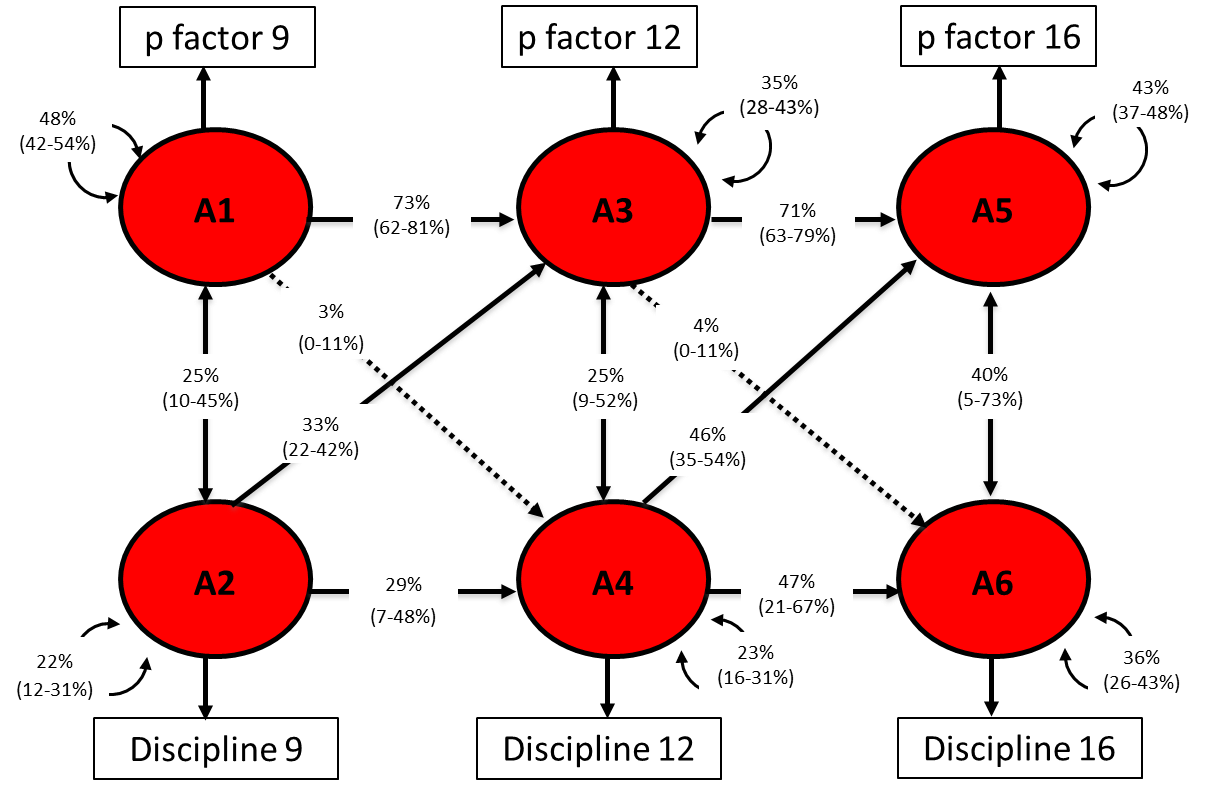
**

**
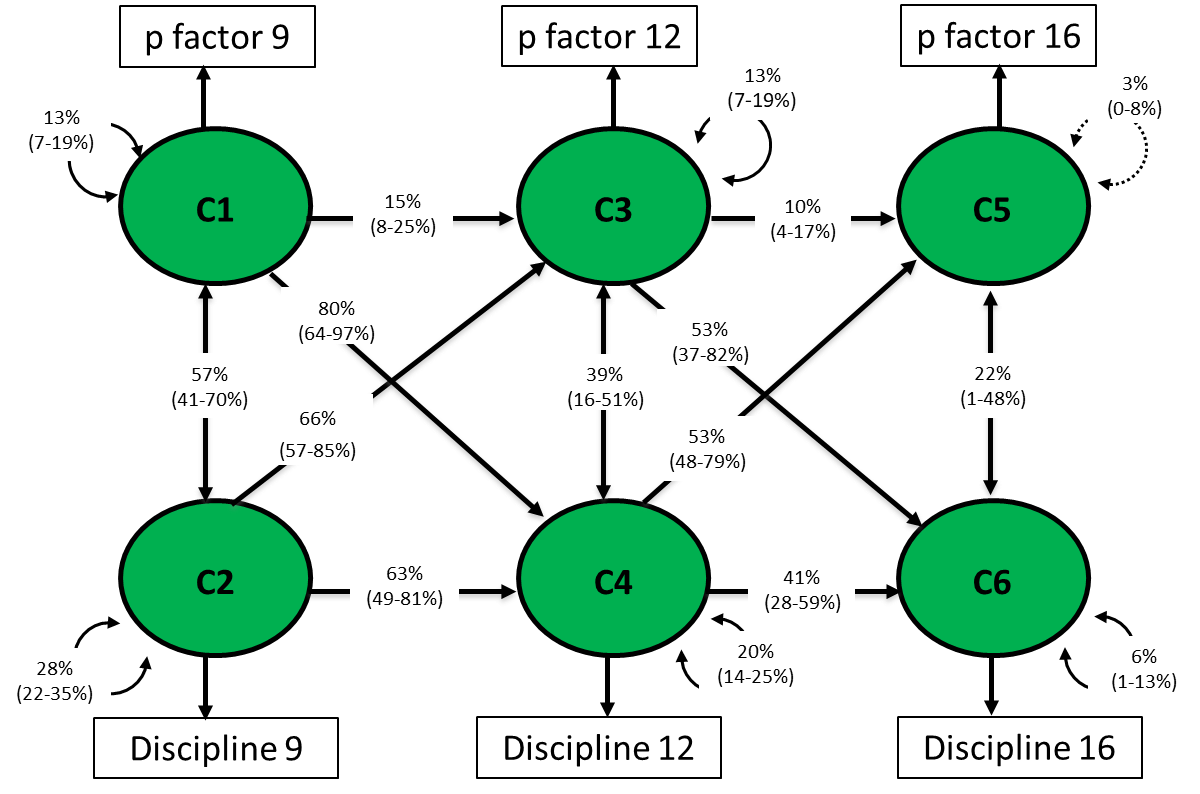
**

**
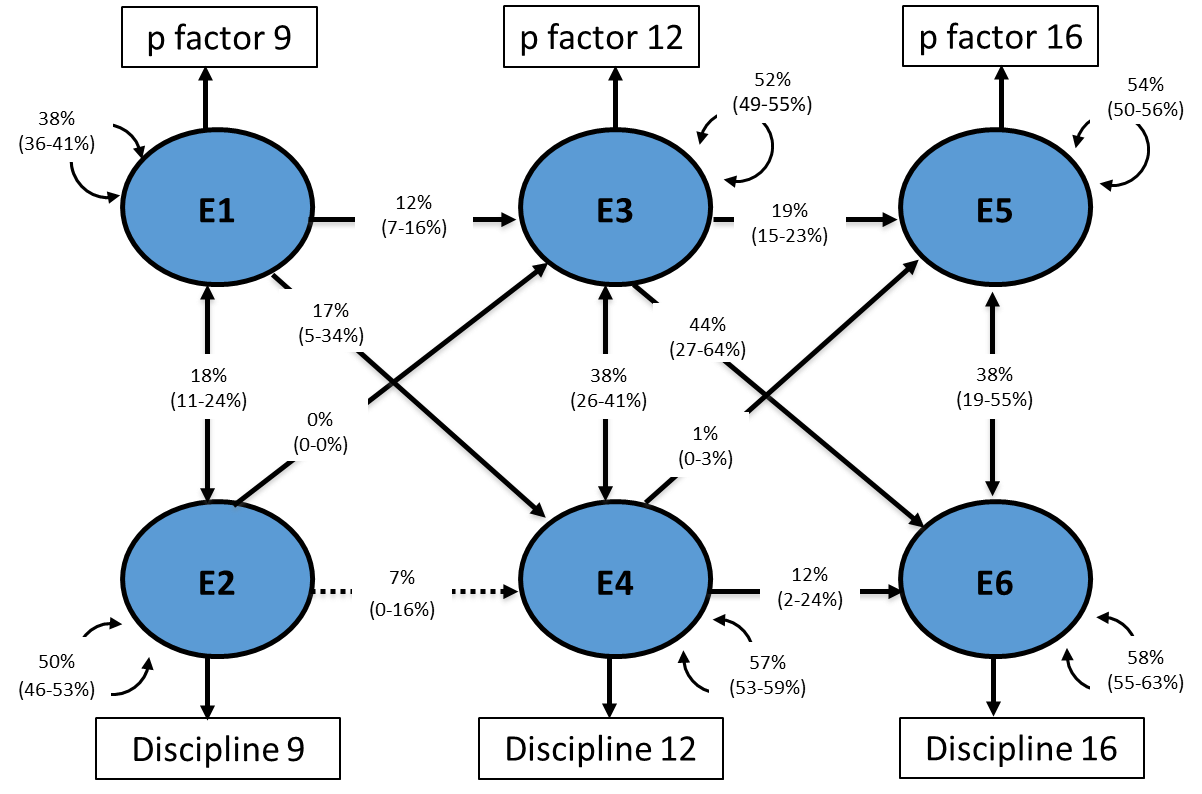
**

## **Figure S13.** *A cross-lagged panel model between the parent-rated p factor (p) and home environment composite (he) MZ difference scores*

0.45

(0.40-0.50)

-0.05

(-0.11-0.00)

0.02

(-0.03-0.08)

0.04

(-0.02-0.10)

0.34

(0.29-0.38)

0.04

(-0.01-0.09)

0.06

(-0.02-0.14)

0.08

(0.01-0.16)

1.00

(1.00-1.00)

1.00

(1.00-1.00)

0.05

(-0.01-0.11)

0.79

(0.75-0.84)

0.88

(0.85-0.91)

1.00

(0.98-1.00)

0.99

(0.97-1.00)

0.07

(0.03-0.11)

0.06

(-0.02-0.13)

Note: 95% CI are in brackets. Solid lines indicate statistical significance; dashed lines indicate non-significance paths estimates

## **Figure S14.** *A cross-lagged panel model between the twin-rated p factor (p) and home environment composite (he) MZ difference scores*

1.00

(1.00-1.00)

1.00

(1.00-1.00)

0.16

(0.09-0.22)

0.09

(0.03-0.16)

0.02

(-0.04-0.08)

0.02

(-0.04-0.08)

0.19

(0.14-0.24)

0.05

(-0.04-0.13)

0.02

(-0.04-0.07)

0.08

(0.00-0.15)

0.18

(0.13-0.23)

0.97

(0.99-1.00)

0.99

(0.97-1.00)

0.97

(0.95-0.99)

0.96

(0.94-0.98)

0.14

(0.08-0.21)

0.26

(0.22-0.30)

Note: 95% CI are in brackets. Solid lines indicate statistical significance where 95% confidence intervals did not cross zero; dashed lines indicate non-significance as a result of crossing zero.

## **Figure S15.** *A cross-lagged panel model between the parent-rated p factor (p) and twin-rated CHAOS MZ difference scores*

0.45

(0.40-0.50)

-0.08

(-0.14--0.02)

0.01

(-0.04-0.07)

0.01

(-0.05-0.07)

0.34

(0.29-0.39)

0.02

(-0.03-0.07)

0.04

(-0.04-0.12)

0.05

(-0.02-0.13)

1.00

(1.00-1.00)

1.00

(1.00-1.00)

0.03

(-0.02-0.09)

0.80

(0.75-0.84)

0.88

(0.85-0.91)

0.99

(0.98-1.00)

1.00

(0.98-1.00)

0.09

(0.05-0.14)

0.06

(-0.01-0.14)

Note: 95% CI are in brackets. Solid lines indicate statistical significance; dashed lines indicate non-significance paths estimates

## **Figure S16.** *A cross-lagged panel model between the twin-rated p factor (p) and twin-rated CHAOS MZ difference scores*

0.16

(0.09-0.22)

0.07

(0.00-0.13)

0.00

(-0.06-0.07)

-0.01

(-0.07-0.06)

0.19

(0.14-0.24)

0.00

(-0.05-0.06)

0.05

(-0.03-0.13)

0.04

(-0.04-0.11)

1.00

(1.00-1.00)

1.00

(1.00-1.00)

0.18

(0.12-0.23)

0.97

(0.95-0.99)

0.96

(0.94-0.98)

1.00

(0.98-1.00)

0.99

(0.98-1.00)

0.25

(0.21-0.29)

0.15

(0.08-0.22)

Note: 95% CI are in brackets. Solid lines indicate statistical significance; dashed lines indicate non-significance paths estimates

## **Figure S17.** *A cross-lagged panel model between the parent-rated p factor (p) and twin-rated parental discipline MZ difference scores*

0.45

(0.40-0.50)

0.00

(-0.06-0.05)

0.02

(-0.04-0.08)

0.05

(-0.01-0.11)

0.34

(0.29-0.39)

0.05

(0.00-0.10)

0.06

(-0.03-0.14)

0.11

(0.03-0.19)

1.00

(1.00-1.00)

1.00

(1.00-1.00)

0.03

(-0.02-0.09)

0.79

(0.75-0.84)

0.88

(0.85-0.91)

1.00

(0.99-1.00)

0.98

(0.96-1.00)

0.02

(-0.02-0.07)

0.12

(0.05-0.20)

Note: 95% CI are in brackets. Solid lines indicate statistical significance; dashed lines indicate non-significance paths estimates

## **Figure S18.** *A cross-lagged panel model between the twin-rated p factor (p) and twin-rated parental discipline MZ difference scores*

0.15

(0.09-0.22)

0.07

(0.01-0.13)

0.04

(-0.02-0.10)

0.05

(-0.02-0.11)

0.19

(0.14-0.24)

0.02

(-0.03-0.07)

0.06

(-0.02-0.14)

0.11

(0.03-0.19)

1.00

(1.00-1.00)

1.00

(1.00-1.00)

0.11

(0.03-0.19)

0.97

(0.95-0.99)

0.96

(0.94-0.98)

0.99

(0.98-1.00)

0.98

(0.96-1.00)

0.15

(0.11-0.19)

0.13

(0.07-0.20)

Note: 95% CI are in brackets. Solid lines indicate statistical significance; dashed lines indicate non-significance paths estimates
